# Plasma proteomic signatures of cellular aging predict human disease

**DOI:** 10.1038/s41591-026-04446-y

**Published:** 2026-06-15

**Authors:** Daisy Yi Ding, Veronica Augustina Bot, Kenneth L. Chen, James W. Groves, Róbert Pálovics, Daisuke Masuda, Amelia Farinas, Hamilton Se-Hwee Oh, Viktoria Wagner, Nannan Lu, The Global Neurodegeneration Proteomics Consortium (GNPC), Daisy Yi Ding, Veronica Augustina Bot, Amelia Farinas, Hamilton Se-Hwee Oh, Carlos Cruchaga, Tony Wyss-Coray, Carlos Cruchaga, Alina Isakova, Jonathan M. Schott, Tony Wyss-Coray

**Affiliations:** 1 Department of Neurology and Neurological Sciences, Stanford University School of Medicine, Stanford, CA USA; 2 The Phil and Penny Knight Initiative for Brain Resilience, Stanford University, Stanford, CA USA; 3 Wu Tsai Neurosciences Institute, Stanford University, Stanford, CA USA; 4 Graduate Program in Bioengineering, Stanford University, Stanford, CA USA; 5 Divisions of Hematology and Oncology, Department of Medicine, Stanford University School of Medicine, Stanford, CA USA; 6 Dementia Research Centre, UCL Queen Square Institute of Neurology, London, UK; 7 Department of Management Science and Engineering, Stanford University, Stanford, CA USA; 8 Graduate Program in Neuroscience, Stanford University, Stanford, CA USA; 9 Nash Family Department of Neuroscience, Icahn School of Medicine at Mount Sinai, New York, NY USA; 10 Brain and Body Research Center of the Friedman Brain Institute, Icahn School of Medicine at Mount Sinai, New York, NY USA; 11 Department of Genetics and Genomic Sciences, Icahn School of Medicine at Mount Sinai, New York, NY USA; 12 Ronald M. Loeb Center for Alzheimer’s Disease, Icahn School of Medicine at Mount Sinai, New York, NY USA; 13 Department of Psychiatry, Washington University, St. Louis, MO USA; 14 NeuroGenomics and Informatics Center, Washington University School of Medicine, St. Louis, MO USA; 15 UK Dementia Research Institute at UCL, London, UK

**Keywords:** Prognostic markers, Alzheimer's disease, Predictive markers, Amyotrophic lateral sclerosis, Neurodegeneration

## Abstract

Aging is asynchronous across cells and organs. Here we tested whether plasma proteomics can be used to analyze cell type-specific aging. From analyses of over 7,000 plasma proteins measured in 60,542 individuals, we developed machine learning models to estimate the biological age of over 40 cell types spanning neuronal, immune, glial, endocrine, epithelial and musculoskeletal origins. We observed that 20–25% of individuals exhibited accelerated aging in a single cell type and 1–3% in 10 or more cell types. Cellular aging signatures were associated with disease status and predicted incident disease and mortality over 15 years of follow-up. Individuals with the *APOE4* genotype showed older astrocytes but younger macrophages compared to *APOE3* carriers, whereas the *APOE2* genotype had inverse associations. Moreover, extreme astrocyte aging tripled the risk of incident Alzheimer’s Disease in individuals with two *APOE4* alleles, while youthful astrocytes reduced risk. Individuals with extremely aged compared to youthful skeletal myocytes exhibited a 12.7-fold higher risk of developing amyotrophic lateral sclerosis. In individuals who smoked, extreme respiratory epithelial cell aging was associated with a 58% higher lung cancer risk compared to smoking alone. Specific cellular vulnerabilities and cumulative cellular aging burden influenced survival, with youthful immune and neuronal cell types conferring protective effects. Finally, we developed a polycellular aging risk score that stratified mortality risk across cohorts and proteomics platforms. These findings establish a framework for quantifying human physiology at cellular resolution, revealing heterogeneous aging trajectories and their impact on disease susceptibility and resilience.

## Abstract (web summary)

The biological age of individual cell types can be evaluated using plasma proteomics, revealing diverse aging profiles across more than 40 cell types and links between the accelerated aging of specific cell types and disease.

## Main

Over 50% of the global disease burden can be attributed to aging^1^. The risk of neurodegenerative disease, cancer and chronic diseases increases sharply with age^2–4^. Despite being the central driver of disease susceptibility, aging itself remains poorly understood, and quantifying its biology is therefore an important priority for improving prevention and treatment.

Recent studies utilizing machine learning models known as aging clocks demonstrate that biological aging varies within and between individuals. Estimators of organ-specific biological age including epigenetic^5^, proteomic^6^, transcriptomic^7^, multiomic^8^ and magnetic resonance imaging-based^9^ aging clocks demonstrate that molecular shifts occur asynchronously across organs throughout the lifespan. Emergent plasma proteomic aging clocks provide a noninvasive window into the aging state of organs, and demonstrate that biologically older organs predispose individuals to organ-specific disease whereas youthful profiles associate with resilience^10,11^. Furthermore, transcriptomic^12,13^ and epigenetic^14^ aging clocks extend these observations to cellular resolution, revealing heterogenous aging patterns across different cell types. While informative, these approaches require harvested tissues or animal models, limiting scalability and translational relevance.

**Fig. 1:**
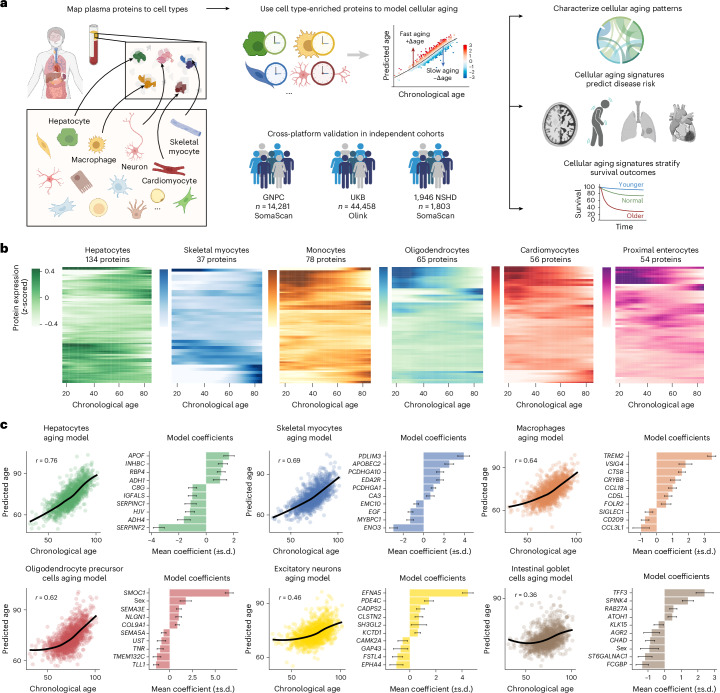
A framework for modeling cellular aging with plasma proteomics. **a**, Study design for evaluating plasma proteomic signatures of cellular aging across platforms and cohorts: plasma proteins were mapped to cell types using the Human Protein Atlas, cell type-enriched proteins were used to train machine learning models that predict chronological age, cell type-specific age gaps were calculated and *z*-scored to enable comparisons across cell types, cross-platform validation was performed in three independent cohorts: the GNPC (*n* = 14,281, SomaScan), the UKB (*n* = 44,458, Olink) and the 1946 NSHD (*n* = 1,803, SomaScan), cellular age gaps were quantified and used to describe heterogeneous aging patterns, predict disease risk and stratify survival outcomes. **b**, Plasma protein expression trajectories for six illustrative cell types in healthy individuals in the GNPC cohort (*n* = 7,074). Trajectories were modeled using LOWESS regression for individuals aged 20 to 90 years, with each plot showing proteins assigned to the indicated cell type. **c**, Illustrative cellular aging models trained on healthy individuals in the Knight-ADRC cohort (*n* = 1,398). Scatter plots show estimated biological age versus chronological age with corresponding correlation coefficients (*r*). Bar plots display mean coefficients (±s.d.) of the top proteins by magnitude in each cellular aging model. Illustrations in **a** created in BioRender; Augustina, V. https://biorender.com/7k7rct4 (2026).

In this study, we perform a comprehensive analysis of cellular aging using plasma measurements from more than 7,000 proteins encompassing 60,542 individuals in three independent cohorts (Fig. 1a). We map plasma proteins to their putative cellular origin and construct computational models that measure the biological age of over 40 cell types. To assess clinical relevance, we quantify age acceleration as age gaps and evaluate associations with disease and mortality.

## Results

**Extended Data Fig. 1:**
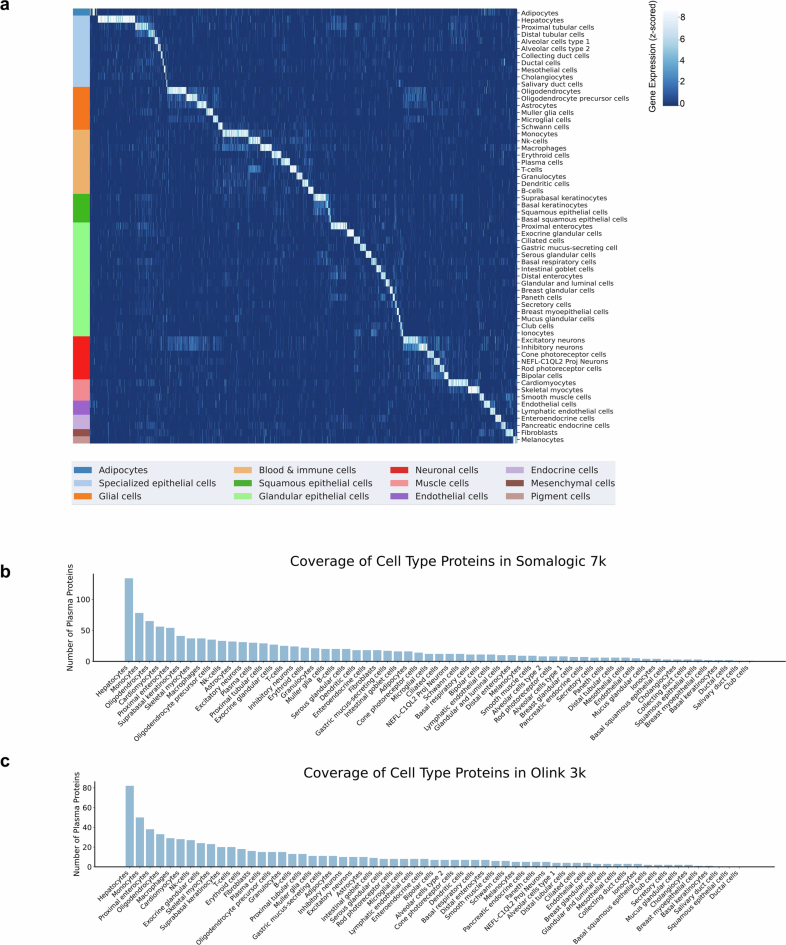
Identification of cell type-specific plasma proteins. **(a)** Z-scored gene expression of cell type-enriched plasma protein encoding genes in the Human Protein Atlas (HPA, version 24.1) single-cell transcriptomics dataset, labeled by cell type and grouped according to HPA cell type family categorizations. **(b)** Coverage of cell type-specific proteins across the 7,289 proteins measured by the 7k SomaScan platform. **(c)** Coverage of cell type-specific proteins across the 2,923 proteins measured by the 3k Olink platform.

**Extended Data Fig. 2:**
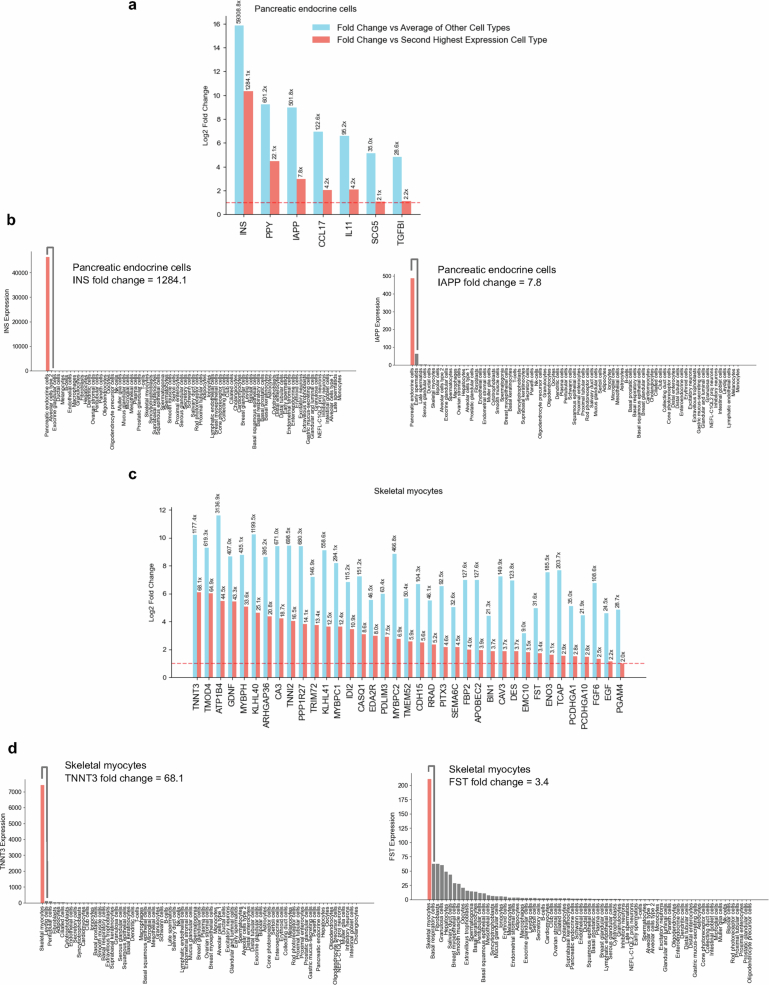
Enrichment profile examples of cell type-specific proteins. **(a)** Fold change of enriched proteins for pancreatic endocrine cells based on the Human Protein Atlas single-cell transcriptomic dataset (version 24.1): blue bars represent fold change compared to average expression across all other cell types; red bars show fold change relative to the second-highest expressing cell type. The y-axis shows fold change on the log2 scale. Numbers above bars indicate specific fold change values. The dashed line represents the threshold used for cell type-specific signatures. **(b)** Expression of two example proteins mapped to pancreatic endocrine cells, INS (left) and IAPP (right), across all cell types. **(c)** The corresponding bar plot of fold change for skeletal myocytes. **(d)** Expression of two example proteins mapped to skeletal myocytes, TNNT3 (left) and FST (right), across all cell types.

Plasma protein abundance is known to vary with age in an organ-specific manner, but whether similar patterns exist at the cellular level remains unknown. Leveraging single-cell transcriptomic data in the Human Protein Atlas (Methods and Extended Data Fig. 1), we linked 60 cell types to their corresponding plasma proteins. Guided by prior studies^10,11,15^, we classified genes as cell-type specific if they were expressed at least twofold higher in one cell type compared to any other cell type. Using this criterion, we found 16.5% of measurable plasma proteins in the SomaScan assay (1,202 out of 7,289) and 24.2% of proteins in the Olink assay (708 out of 2,923) mapped to specific cell types (Supplementary Tables 1 and 2). Although the twofold enrichment threshold may seem permissive, many proteins mapped to cell types have fold changes markedly exceeding this criterion, supporting the premise that cell types possess unique signatures that are detectable in plasma (Methods and Extended Data Fig. 2). We observed distinct age-related plasma protein expression patterns across different cell types, as illustrated for 7,074 healthy individuals in the Global Neurodegeneration Proteomics Consortium (GNPC), a large-scale international neurodegenerative disease plasma proteomics resource comprising multiple subcohorts^16^ (Fig. 1b and Supplementary Table 3).

**Extended Data Fig. 3:**
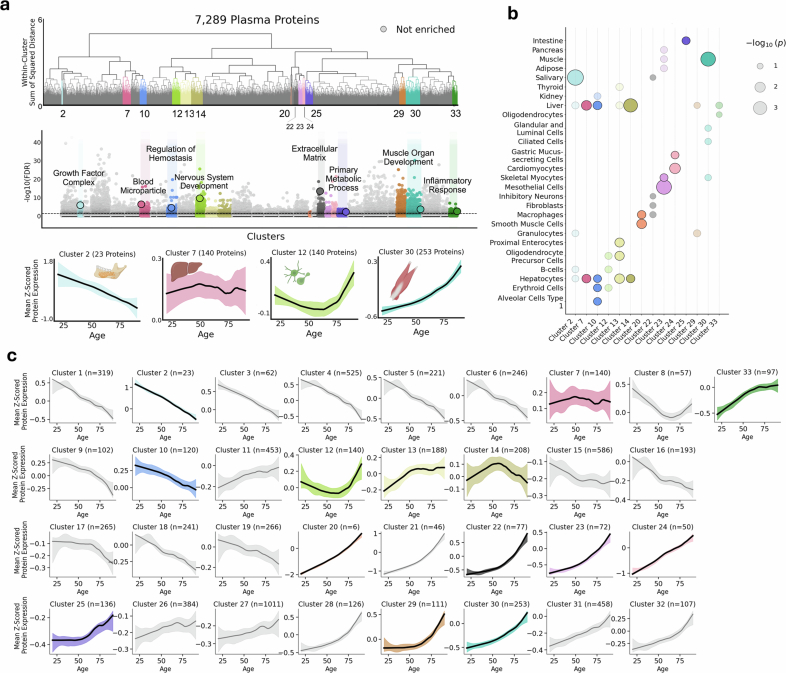
Clustering of plasma protein trajectories. **(a)** Expression of 7,289 plasma proteins was measured in 7,074 healthy individuals in the GNPC cohort, plotted against chronological age using locally weighted scatterplot smoothing (LOWESS) regression. Protein trajectories were clustered using hierarchical clustering, resulting in 33 distinct clusters. Colored clusters indicate significant enrichment for one or more cell types and/or organs. Gray clusters indicate no significant enrichment. For each cluster, the central curve represents the mean LOWESS trajectory of *z*-scored protein expression across chronological age, and shaded bands indicate 95% confidence intervals (CIs) around the mean estimate. **(b)** Cell-type trajectory clustering enrichment among healthy individuals (n = 7,074). Associations were assessed using two-sided Fisher’s exact tests, with *P* values adjusted for multiple comparisons using the Benjamini–Hochberg procedure. Translucent dots indicate adjusted *P* < 0.1, and opaque dots indicate adjusted *P* < 0.05. **(c)** All 33 clusters are shown. For each cluster, the central curve represents the mean LOWESS-smoothed trajectory of z-scored protein expression across age, and shaded bands indicate 95% confidence intervals around the mean estimate. A total of 7,074 individuals were included. Illustrations in **a** created in BioRender; Augustina, V. https://biorender.com/7k7rct4 (2026).

Building on these observations, we performed unsupervised hierarchical clustering of plasma protein expression patterns to identify groups of proteins whose age-related changes occur in concert. We identified 33 clusters ranging in size from 6 to 1,011 proteins that increased, decreased or remained stable over time (Extended Data Fig. 3). Clusters exhibited significant and interpretable enrichment for cell types and biological pathways, spanning hepatocytes and regulation of coagulation (Cluster 10, adjusted *P* = 8.32 × 10^−6^; Supplementary Table 4), to B cells, oligodendrocyte precursor cells and nervous system development (Cluster 12, adjusted *P* = 2.49 × 10^−10^; Supplementary Table 4). Intriguingly, we found that cells of a shared lineage co-occur within clusters (for example endoderm-derived ciliated cells, glandular and luminal cells or mesoderm-derived mesothelial cells and skeletal myocytes); a discovery that may reflect developmental shifts during lifespan and coordinated gene expression programs^17,18^. The stability of clusters was evaluated by performing a sensitivity analysis of trajectory clustering with bootstrap resampling (Supplementary Fig. 1), revealing our results to be robust.

These observations support our central hypothesis that cell types age at different rates, and their aging trajectories can be captured through variations in plasma protein abundance. We next built population-based models of biological aging for different cell types by training machine learning models to predict chronological age based on the plasma abundance of cell type-specific proteins (Methods). To demonstrate robustness and generalizability, we developed cellular aging clocks for two plasma proteomics platforms—SomaScan (measuring 7,289 proteins) and Olink (measuring 2,923 proteins)—and applied the models in three independent cohorts: the GNPC (*n* = 14,281, SomaScan), the 1946 National Survey of Health and Development (NSHD; *n* = 1,803, SomaScan) and the UK Biobank (UKB; *n* = 44,458, Olink).

For the SomaScan platform, models were trained on plasma protein data generated from healthy individuals in the Knight Alzheimer’s Disease Research Center (KADRC) cohort, the largest well-characterized healthy cohort in the GNPC. SomaScan models were applied to the broader GNPC cohort for disease association analyses, and to the NSHD cohort for external validation. We generated aging models for over 60 different cell types, with 43 models retained for downstream analysis after performance quality assessment (Methods; see Supplementary Tables 5–7 for a list of retained models across proteomics platforms). Model performance in the KADRC cohort is visualized in Fig. 1c and Supplementary Fig. 2.

Using a similar approach, cellular aging models were built with Olink plasma proteomics using a training subset of the UKB cohort (*n* = 21,983) and applied to a held-out test set in the UKB (*n* = 22,475). Given that the Olink platform measures fewer proteins and thus fewer cell type-specific signatures, we additionally incorporated 14 lineage-level cell-type models that aggregate ontologically related cell types (for example, lymphoid lineage combining B cells, T cells, NK cells and plasma cells) to capture aging signatures across platforms and gain insight into cellular aging at varying levels of granularity (Supplementary Table 5, Supplementary Fig. 3 and Methods). After performance quality assessment (Methods), 48 Olink cellular aging models were retained for downstream analysis. The resulting models were comprised of plasma proteins uniquely linked to each cell type (Supplementary Fig. 2 and Supplementary Tables 5–7).

For each cell type and individual, we calculated an ‘age gap’, defined as the residual between the individual’s predicted cell-type-specific biological age and the model-predicted biological age of an average individual of the same chronological age. Positive age gaps indicate accelerated aging, while negative gaps represent relative biological youth. Age gaps were *z*-scored per aging model to facilitate comparison across cell types. For each cell type, individuals were categorized based on their *z*-scored age gap, wherein extreme aging and youthful aging were defined as having a *z*-scored age gap >2 and <−2, respectively^11^.

**Fig. 2:**
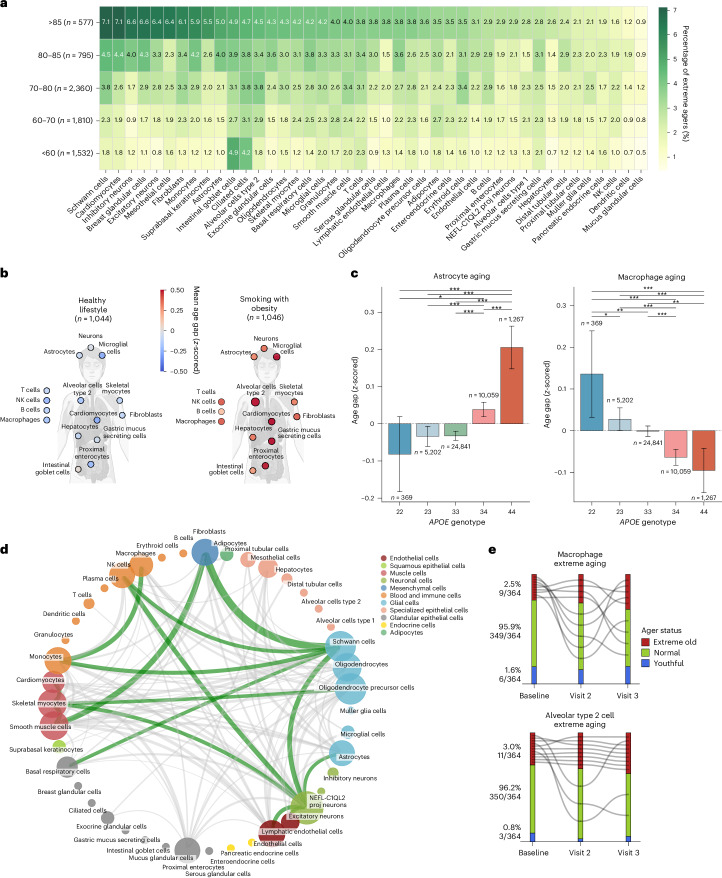
Cellular aging signatures reveal individual heterogeneity. **a**, Age-dependent patterns of extreme cellular aging in healthy individuals within the GNPC cohort (*n* = 7,074). Heatmap shows the percentage of extreme agers across 43 cell types (columns) and five chronological age windows (rows), with extreme agers defined as individuals with age gap at least two s.d. from the mean. **b**, Cellular aging and modifiable risk factors in the UKB cohort. Representative cellular aging profiles for individuals with a healthy lifestyle (left: *n* = 1,044) defined as never smoking, no regular alcohol consumption, at least 5 days per week of 10+ min of moderate or vigorous physical activity, BMI < 25, waist circumference <90 cm for men or <84 cm for women, ≥7 h of sleep nightly, and individuals with concurrent smoking and obesity (right: *n* = 1,046). Color intensity corresponds to the mean *z*-scored age gap. **c**, Bar plots show mean *z*-scored age gaps for astrocytes (left) and macrophages (right) stratified by *APOE* genotype (*APOE2/2*: *n* = 369, *APOE2/3*: *n* = 5,202, *APOE3/3:* *n* = 24,841, *APOE3/4*: *n* = 10,059, *APOE4/4*: *n* = 1,267) in the UKB cohort. Data are presented with error bars indicating 95% CI. Statistical significance between *APOE* genotype groups was assessed using two-sided independent *t*-tests. *P* values are indicated as ^*^P < 0.05, ^**^P < 0.01 and ^***^P < 0.001. **d**, Correlation network showing cellular aging patterns across cell types in healthy individuals in the GNPC cohort (*n* = 7,074). Each node represents a specific cell type, with two nodes connected if the correlation of age gaps between the two cell types is above a threshold of 0.35. Edge width corresponds to correlation strength, with edges highlighting the top 15 correlations. **e**, Stability of extreme cellular aging over a 10-year period for individuals categorized as macrophage extreme agers (top: *n* = 9, *z*-scored age gap >2) and alveolar type 2 cell extreme agers (bottom: *n* = 11, *z*-scored age gap >2) in the NSHD cohort (timepoints: 63.2 ± 1.1 years (baseline), 70.7 ± 0.7 years (visit 2), 72.9 ± 0.6 years (visit 3), *n* = 364 across all timepoints). Age ranges are represented as mean ± s.d years. Bars show the proportion of extreme old, extreme young and normal agers at each timepoint, with extreme groups proportionally scaled for visualization. Illustrations in **b** created in BioRender; Augustina, V. https://biorender.com/7k7rct4 (2026).

To describe normative cell-type biological aging patterns, we applied SomaScan cellular aging models to 7,074 healthy individuals in the GNPC cohort and visualized the distribution of extreme agers across five chronological age windows (Fig. 2a). Notably, neuronal and glial cell types—including Schwann cells, inhibitory and excitatory neurons—showed elevated age gaps later in life, with 7.1%, 6.6% and 6.4% of individuals classified as extreme agers in the over 85-year-old age group. In contrast, intestinal goblet cells and ciliated cells exhibited early onset of accelerated aging, affecting 4.9% and 4.2% of individuals under 60 years old. We hypothesize that the prevalence of cell-specific biological extreme agers in unique chronological age windows could reflect cellular vulnerabilities and the timing of disease onset. While accelerated aging of neurons and Schwann cells with advanced age may be related to cognitive impairment and loss of sensory perception with age, accelerated aging of goblet and ciliated cells in younger individuals may point to increased gut leakiness and reduced ependymal cilia barrier integrity during midlife^19–21^.

**Extended Data Fig. 4:**
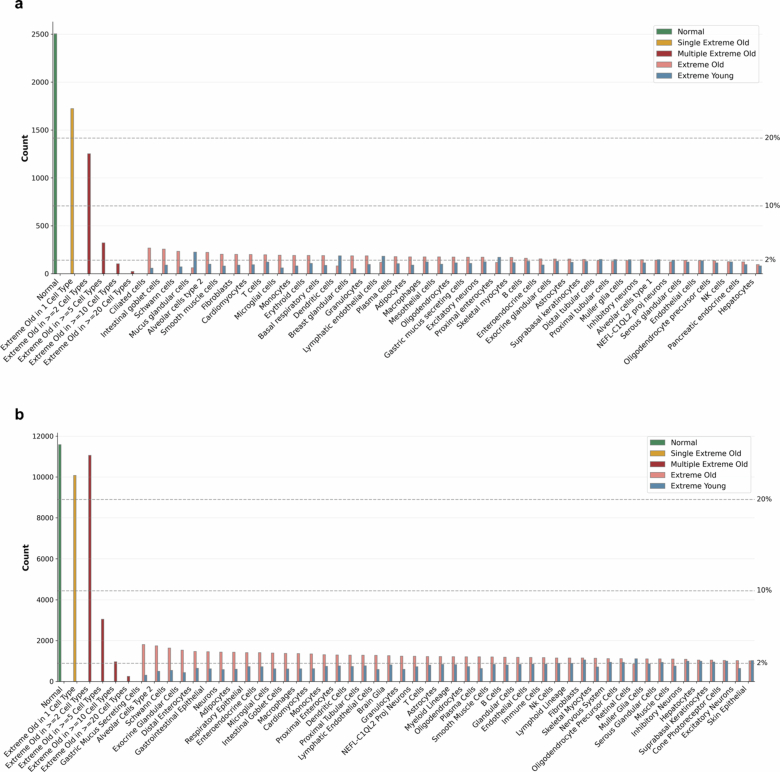
Distribution of extreme aging profiles. **(a)** Distribution of extreme aging profiles across cell types among healthy individuals in the GNPC cohort (n = 7,074). The bar plot shows the count of individuals categorized by aging status: normal (green, individuals without extreme aging in any cell type), single extreme aging (orange, extreme aging in only one cell type), multiple extreme aging (dark red, extreme aging in multiple cell types), extreme aging by specific cell type (pink, z-scored age gap > 2 for the indicated cell type), and decelerated aging by specific cell type (blue, z-scored age gap < −2 for the indicated cell type). Horizontal dashed lines indicate reference points at 2%, 10%, and 20% of the cohort. **(b)** Distribution of extreme aging profiles across cell types in the UKB cohort (n = 44,458). All categorizations, color schemes, and reference lines are as described in **(a)**.

Across all healthy individuals in the GNPC cohort, we found 35.4% had no extreme cellular age gaps and 24.4% had accelerated aging in a single cell type, while 1.5% of the population experienced widespread acceleration across 10 or more cell types. For each cell type studied, we found 0.9–3.8% of the population had extremely old cells, and 0.7–3.2% had extremely young cells (Extended Data Fig. 4a). Similarly, in the UKB, 26.1% showed no extreme cellular age gaps, 22.7% had accelerated aging in a single cell type, and 2.8% exhibited widespread acceleration across 10 or more cell types, with 1.9–4.1% showing extreme acceleration and 0.7–2.5% showing extremely young cells per cell type (Extended Data Fig. 4b).

Notably, cellular age gaps demonstrated associations with modifiable risk factors in the UKB cohort. Among individuals with concurrent smoking and obesity (*n* = 1,046), we observed widespread increase in biological age across multiple cell types, while individuals with a healthy lifestyle (*n* = 1,044) defined as never smoking, no alcohol consumption, body mass index (BMI) lower than 25 without enlarged waist circumference, sufficient sleep (≥7 h nightly) and regular exercise (≥5 days weekly), showed overall younger cellular ages (Fig. 2b).

Our analysis of genetic influences in the UKB cohort demonstrates that specific genotypes accelerate aging in certain cell types while preserving others (Fig. 2c). The *APOE* genotype, a major risk factor for neurodegenerative disease, showed dose-dependent, antagonistic effects on immune versus central nervous system cell aging: *APOE2* carriers exhibited significantly younger astrocyte profiles but older macrophages, while *APOE4* carriers showed the inverse, with older astrocytes but younger macrophages, suggesting antagonistic pleiotropy operating at cellular resolution. This aligns with the evolutionary hypothesis that *APOE4*’s enhanced immune vigilance conferred survival advantages under historically high pathogen burdens, despite accelerated brain aging that increases Alzheimer’s disease (AD) risk in modern extended lifespans^22,23^.

The examination of co-occurring age gap profiles across 7,074 healthy individuals in the GNPC cohort suggests cellular aging may proceed in a concerted fashion across a small number of cell types (Fig. 2d). These coordinated patterns were particularly pronounced among excitatory neurons, myelinating cells and endothelial cells, suggesting shared or synchronized pathways. Certain cell populations—such as excitatory neurons, Schwann cells, NK cells, macrophages, skeletal myocytes and fibroblasts—emerged as potential ‘aging hubs’, showing correlations with multiple other cell types. In contrast, epithelial cell types tended to exhibit more isolated or weakly correlated age gap profiles.

By analyzing the NSHD, the world’s longest continuously followed birth cohort^24^, we examined the stability of extreme cellular aging in 364 individuals over a 10-year period. In Fig. 2e, we show two representative profiles: macrophage extreme agers (9 of 364 individuals at baseline), and alveolar type 2 cell type extreme agers (11 of 364 individuals at baseline). There was substantial retention of extreme aged states: 55% of baseline macrophage extreme agers retained this status during the 10-year follow-up period, whereas alveolar type 2 cell extreme agers showed 81% retention. We extended this analysis to baseline youthful cell-type agers in Supplementary Fig. 4a, revealing the presence of unique stability profiles across different cell types (Supplementary Fig. 4a,b). Our observations suggest that individuals in a state of extreme cellular aging tend to retain this state over time. Certain cell types show maintenance of old or young states, potentially reflecting differential susceptibility to biological aging^25^.

Taken together, these analyses illustrate that plasma-derived, cell-type specific biological age estimates are linked to heterogeneous cellular aging patterns, with potential implications for disease susceptibility and resilience.

**Extended Data Fig. 5:**
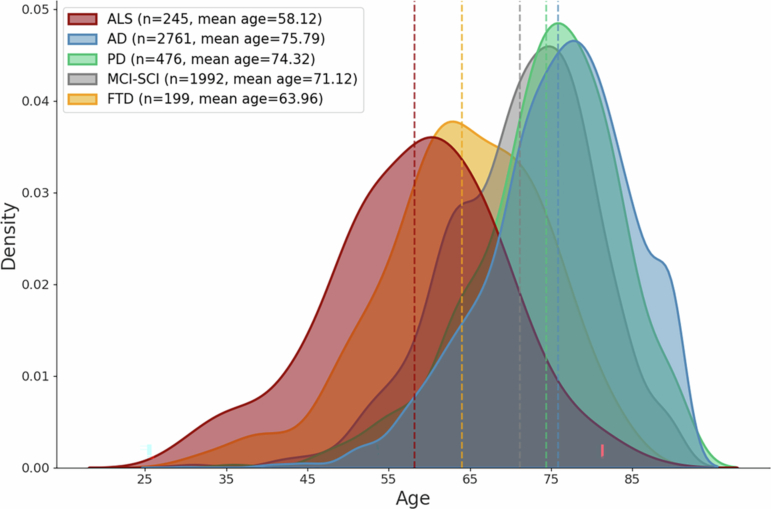
Age distribution across neurodegenerative diseases in GNPC. Density plot showing the chronological age distribution across the five neurodegenerative disease groups in GNPC cohort. ALS (mean=58.12 years, s.d.=10.94), FTD (mean=63.96 years, s.d.=10.14), AD (mean=75.79 years, s.d.=8.83), PD (mean=74.32 years, s.d.=8.84), and MCI-SCI (mean=71.12 years, s.d.=9.11).

Meaningful biological age estimates should capture underlying physiological states and associate with health trajectories and disease outcomes. Thus, we assessed whether cellular age gaps correlate with disease status, focusing on neurodegenerative conditions present in the GNPC: AD (*n* = 2,761), amyotrophic lateral sclerosis (ALS, *n* = 245), Parkinson’s disease (PD, *n* = 476), frontotemporal dementia (FTD, *n* = 199) and mild cognitive impairment–subjective cognitive impairment (MCI–SCI, *n* = 1,992). The chronological age distribution of patients with these five neurodegenerative conditions in the GNPC is illustrated in Extended Data Fig. 5.

**Fig. 3:**
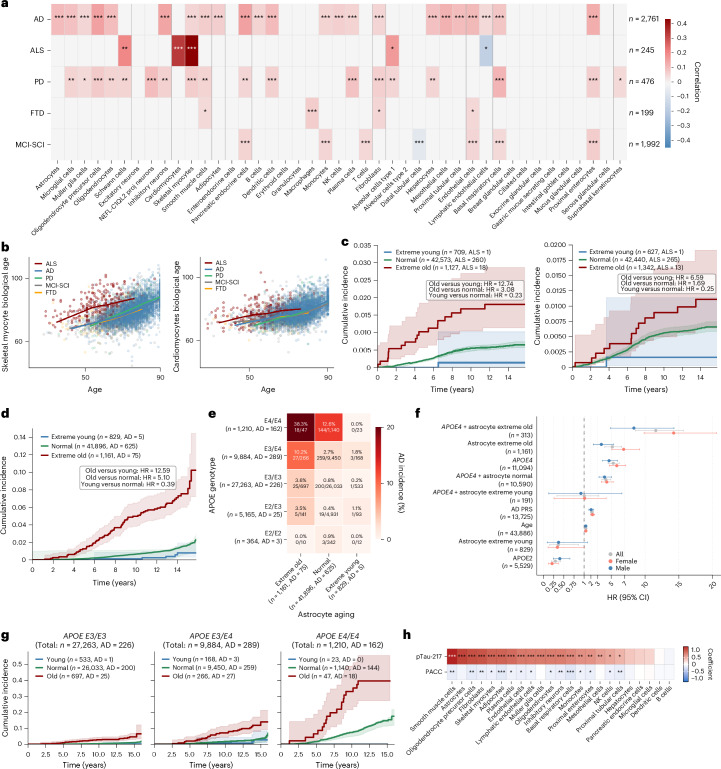
Cell type-specific age estimates are associated with neurodegenerative diseases. **a**, Correlation between cell type-specific age gaps and neurodegenerative disease diagnosis in the GNPC cohort. Analysis includes AD (*n* = 2,761), ALS (*n* = 245), PD (*n* = 476), FTD (*n* = 199) and MCI–SCI (*n* = 1,992). Associations with adjusted *P* values below 0.01 and correlation magnitudes greater than 0.05 are shown in color, with color intensity corresponding to correlation strength, and significance levels annotated. Associations between continuous cellular age gaps and binary disease status were assessed using two-sided point-biserial correlation, with *P* values adjusted using the Benjamini–Hochberg procedure (^*^P < 0.01, ^**^P < 0.001 and ^***^P < 0.0001). **b**, Estimated biological age of skeletal myocytes (left) and cardiomyocytes (right) versus chronological age by neurodegenerative disease diagnosis in the GNPC cohort. Each dot represents an individual, colored by disease diagnosis. A locally weighted scatterplot smoothing (LOWESS) regression fit was used to estimate the relationship between estimated biological and chronological age for each group. **c**, Cumulative incidence of ALS over 15 years of follow-up in the UKB cohort, stratified by skeletal myocyte (left) and cardiomyocyte (right) aging status. Extreme, normal and youthful agers are shown, with sample sizes in the legend. Lines represent Kaplan–Meier estimates, and shaded bands indicate 95% CIs. Overall log-rank test comparing cumulative incidence across youthful, normal and extreme ager groups: skeletal myocyte aging, *P* < 0.0001, cardiomyocyte aging: *P* = 0.0562. **d**, AD cumulative incidence by astrocyte aging status over 15 years of follow-up in the UKB cohort: extreme agers (*n* = 1,161), normal agers (*n* = 41,896) and youthful agers (*n* = 829). Lines represent Kaplan–Meier estimates of cumulative incidence, and shaded bands indicate 95% CIs. Overall log-rank test comparing cumulative incidence across extreme young, normal and extreme ager groups: *P* < 0.0001. **e**, AD cumulative incidence stratified by *APOE* genotype and astrocyte aging status over 15 years of follow-up in the UKB cohort. Rows: *APOE* genotype (*APOE2/2:* *n* = 364, *APOE2/3*: *n* = 5,165, *APOE3/3*: *n* = 27,263, *APOE3/4*: *n* = 9,884, *APOE4/4*: *n* = 1,210). Columns: astrocyte aging (extreme: *n* = 1,161, normal: *n* = 41,896, youthful *n* = 829). Color intensity indicates the cumulative incidence percentage, with numbers displayed in each cell indicating the total number of individuals and the number who developed AD during follow-up. **f**, HRs with 95% CIs for incident AD by risk factor in the UKB cohort. HRs correspond to exponentiated model coefficients, and horizontal lines denote 95% CIs. Overall and sex-stratified results are shown, with sample sizes indicated below each label. **g**, AD cumulative incidence by astrocyte aging status across *APOE* genotypes in the UKB cohort for extreme, normal and youthful agers. Left: *APOE3/3*: *n* = 27,263, middle: *APOE3/4*: *n* = 9,884, right: *APOE4/4*: *n* = 1,210. Lines represent Kaplan–Meier estimates of cumulative incidence, and shaded bands indicate 95% CIs. Within each *APOE* genotype stratum, group differences were assessed using a two-sided log-rank test comparing the extreme astrocyte aging group with the combined young and normal ager groups: *APOE3/3*: *P* < 0.0001, *APOE3/4*: *P* < 0.0001, *APOE4/4*: *P* < 0.0001. **h**, Cellular age gap associations with plasma pTau-217 burden (pg ml^−1^) and PACC (a composite measure of cognitive performance) in the NSHD cohort Insight-46 substudy (*n* = 483). Associations were assessed using linear regression models adjusted for chronological age and sex. For analyses involving PACC, models were additionally adjusted for childhood cognitive ability to account for baseline differences in cognition. Effect sizes are reported as regression coefficients, with two-sided *P* values derived from *t*-statistics and corrected for multiple testing using the Benjamini–Hochberg procedure (*P < 0.05, **P < 0.01 and ***P < 0.001). Lower PACC scores indicate worse cognitive performance.

Utilizing SomaScan aging models, we tested associations between all 43 cell-type age gaps and each neurodegenerative disease using point-biserial correlation with Benjamini–Hochberg false discovery rate correction (Fig. 3a). The strongest association among all disease-cell type pairs was between ALS and skeletal myocyte aging (*r* = 0.43, adjusted *P* = 1.36×10^−15^), consistent with known pathophysiological motor neuron degeneration and muscle atrophy in ALS^26,27^.

Interestingly, along with skeletal myocytes, we observed that cardiomyocytes showed accelerated aging in patients with ALS (*r* = 0.33, adjusted *P* = 4.08 × 10^−9^), consistent with emerging evidence of cardiac abnormalities in patients with ALS^28^, and potentially driven by shared molecular pathways affecting both cardiac and skeletal muscle tissues. Given the strong association between ALS and both skeletal myocyte and cardiomyocyte aging, we visualized the estimated biological ages for these cell types versus chronological age across disease groups (Fig. 3b). Locally weighted scatterplot smoothing (LOWESS) regression analysis revealed that patients with ALS exhibited pronounced acceleration of skeletal myocyte aging and cardiomyocyte aging across chronological age span.

AD was associated with accelerated aging across a wide range of cell types, most prominently oligodendrocyte precursor cells (*r* = 0.15, adjusted *P* = 1.86 × 10^−44^), pancreatic endocrine cells (*r* = 0.15, adjusted *P* = 3.49 × 10^−44^), inhibitory neurons (*r* = 0.15, adjusted *P* = 1.48 × 10^−40^), proximal enterocytes (*r* = 0.13, adjusted *P* = 3.85 × 10^−33^) and astrocytes (*r* = 0.10, adjusted *P* = 3.25 × 10^−21^). Of interest, while aging of inhibitory neurons was linked to AD, aging of excitatory neurons was not (Fig. 3a), potentially supporting a growing body of literature implicating network hyperexcitability related to loss of synaptic inhibition and selective vulnerability of inhibitory interneurons in the pathophysiological process^29,30^. Notably, while we recognize that AD comorbidities such as diabetes may introduce confounding effects, stratified analyses revealed that patients with AD exhibited accelerated pancreatic endocrine cell aging in the absence of type 2 diabetes, while type 2 diabetes amplified this effect in a compounding manner (Supplementary Fig. 5). These findings highlight the systemic nature of AD pathophysiology, with particularly strong connections to gut epithelial aging, metabolic dysregulation and oligodendroglial-neuronal interactions^31–33^.

To further prioritize cell types linked with neurodegenerative diseases, we investigated odds ratios (OR) between cell type-specific extreme ager status and disease states (Supplementary Fig. 6). ALS exhibited an exceptionally strong OR with accelerated skeletal myocyte aging (OR = 7.85, adjusted *P* = 5.50 × 10^−5^), indicating that individuals with extreme skeletal myocyte aging were >7 times more likely to have ALS than those without acceleration. In the ALS cohort within the GNPC cohort (*n* = 355; 245 patients with ALS, 110 controls), 53 of 57 (93.0%) skeletal myocyte extreme agers had an ALS diagnosis. This pronounced association is consistent with reported muscle pathology and emerging evidence that peripheral tissues may contribute to disease progression alongside motor neuron degeneration in ALS^34,35^.

Notably, FTD showed strong cross-sectional association with the cell type initially labeled as ‘horizontal cell’ in the Human Protein Atlas, which, to accurately reflect the biological underpinning of this aging signature, we refer to as NEFL-C1QL2 projection neuron aging (Supplementary Fig. 7). NEFL is a widely recognized biomarker of axonal injury, often markedly raised in FTD, while C1QL2 is a synaptic organizer known to be prominent in temporo-limbic structures vulnerable to frontotemporal lobar degeneration^36,37^. Collectively, these disease-patterned cellular aging signatures support several plausible pathophysiological associations and may also highlight potential new peripheral cellular targets for mechanistic study and therapeutic intervention.

While the cross-sectional study described reveals associations between cellular aging and disease status, a critical question is whether cellular aging is predictive of future disease onset. To address this question, we examined whether cellular aging signatures can stratify risk of incident neurodegenerative disease in the UKB cohort over 15 years of follow-up.

**Extended Data Fig. 6:**
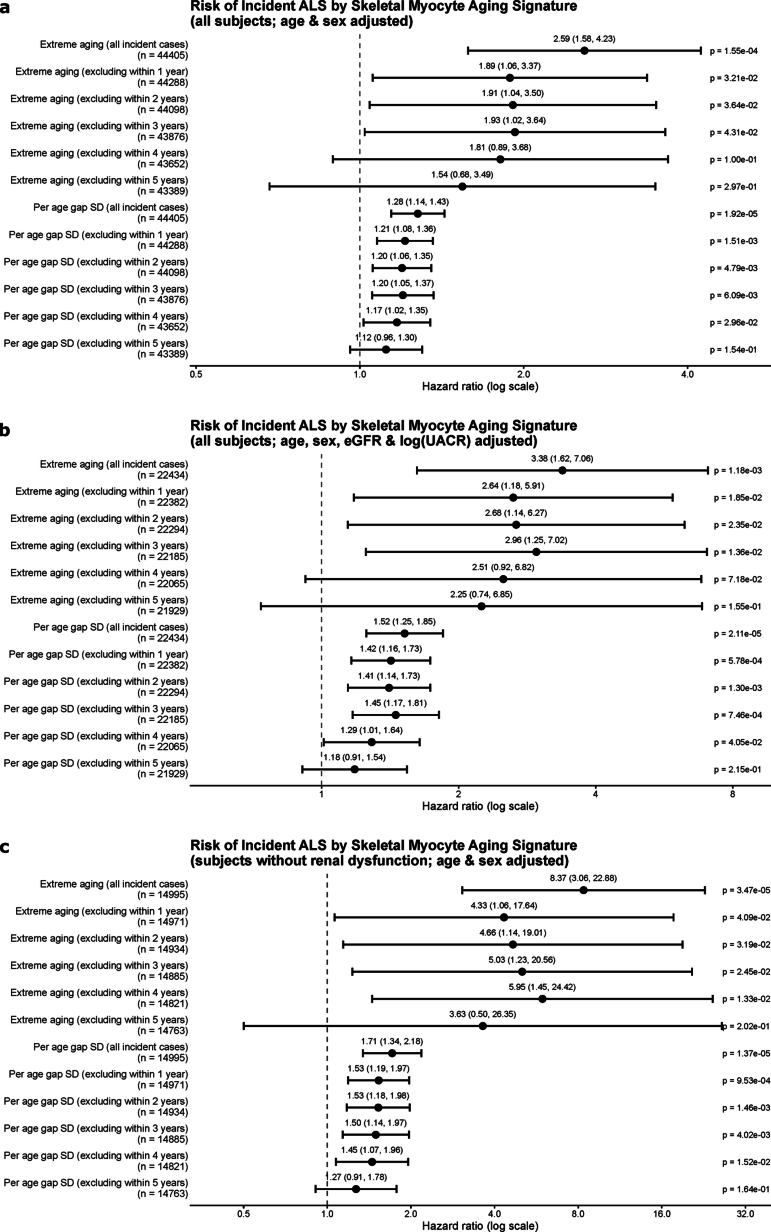
Skeletal myocyte aging has prognostic power for incident ALS. **(a)** Forest plot showing hazard ratios corresponding to the association between incident ALS and skeletal myocyte aging, evaluated with respect to both extreme aging status and continuous age gaps (z-scored). Models include all incident ALS cases, or exclude cases diagnosed within 1, 2, 3, 4, or 5 years after plasma collection to assess predictive power across lag periods. **(b)** HRs with additional adjustment for estimated glomerular filtration rate (eGFR) and log-transformed urine albumin-to-creatinine ratio (UACR). **(c)** Hazard ratios in a subpopulation restricted to individuals without evidence of kidney dysfunction (defined as eGFR ≥ 90 mL/min/1.73m2 and normal (A1) urine albumin-creatinine ratio (UACR); no adjustment for renal function variables. All error bars indicate 95% CIs for Cox proportional hazards models, with baseline adjustment for chronological age and sex.

For ALS, individuals with extreme skeletal myocyte aging exhibited a substantially increased risk of incident ALS compared to those with youthful aging (hazard ratio (HR) = 12.74). Kaplan–Meier analyses across aging groups (extreme, normal and youthful) demonstrated significant differences in cumulative ALS incidence (Fig. 3c; log-rank *P* < 0.0001). Interestingly, the relationship between accelerated skeletal muscle aging and future ALS diagnosis persists even when only considering cases diagnosed more than 3 years after blood draw and cellular aging assessment (Extended Data Fig. 6). Time to diagnosis for ALS is often delayed into the range of 8–15 months, but the multiyear gap could suggest pathological mechanisms affecting muscle tissue begin years before symptom onset^38^. We observed similar risk stratification for cardiomyocyte aging and incident ALS (Fig. 3c; extreme versus youthful aging: HR = 6.59).

When examining the prognostic value of cellular aging signatures for incident AD risk, astrocyte aging emerged as the strongest predictor (Fig. 3d). Individuals with extreme astrocyte aging demonstrated a 12.59-fold increased risk of incident AD compared to those with youthful aging. Kaplan–Meier analyses revealed significant risk stratification by aging status (log-rank *P* < 0.0001), which remained robust across *APOE* genotype subgroups (Fig. 3g).

Given our previous finding that astrocytes showed accelerated aging in *APOE4* carriers in a dose-dependent manner (Fig. 2c), we next examined how *APOE* genotype and astrocyte aging jointly influence AD incidence. Analysis of cumulative incidence across all combinations of *APOE* genotype and astrocyte aging status revealed pronounced synergistic effects (Fig. 3e). Individuals who were homozygous for *APOE4* and had extreme astrocyte aging showed the highest cumulative incidence of 38.3% over 15 years of follow-up, compared to 12.6% for homozygotes with normal astrocyte aging. This risk gradient was consistent across genotypes: *APOE3/4* carriers with extreme astrocyte aging showed 10.2% cumulative incidence versus 2.7% with normal astrocyte aging, and *APOE3/3* carriers demonstrated a similar pattern (3.6% versus 0.8%, respectively).

Of potential therapeutic relevance, none of the 23 *APOE4/4* carriers with youthful astrocytes and only 1.8% of *APOE3/4* carriers with youthful astrocytes developed AD. While only 10 *APOE2/2* carriers exhibited extreme astrocyte aging, *APOE2/2* carriers maintained low incidence overall, consistent with the known protective effects of the *APOE2* allele. These results potentially implicate central ‘inflammaging’ mechanisms and astrocyte activation as key to understanding why only some *APOE4* carriers are susceptible to AD^39,40^, while others remain protected.

**Extended Data Fig. 7:**
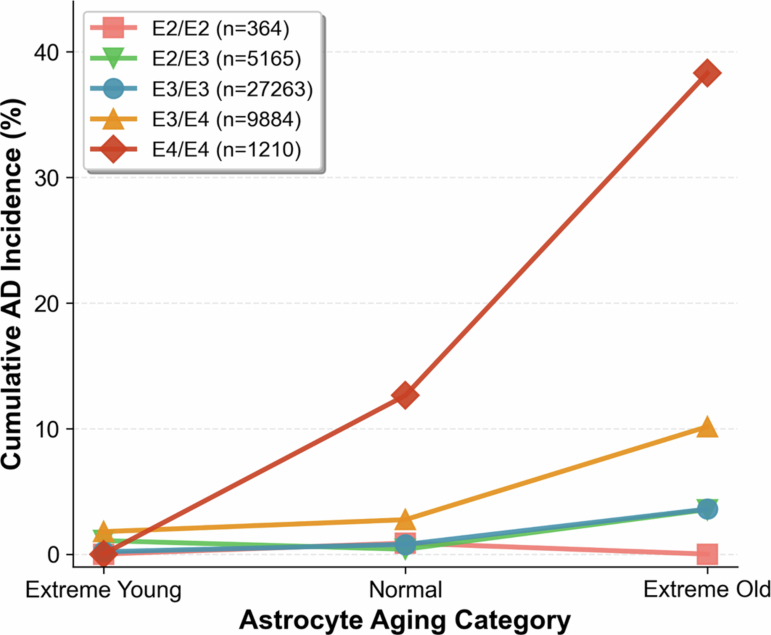
Cumulative AD incidence over 15 years of follow-up in the UKB cohort by astrocyte aging status (extreme young, normal, extreme old) and *APOE* genotype. Lines illustrate the cumulative incidence of Alzheimer’s disease (AD) (%) by astrocyte aging status and *APOE* genotype: *APOE2/2* (n = 364), *APOE2/3* (n = 5,165), *APOE3/3* (n = 27,263), *APOE3/4* (n = 9,884), and *APOE4/4* (n = 1,210). Points represent cumulative incidence estimates within each group.

Consistent with the known genetic risk gradient for AD, disease incidence progressively increased from *APOE3/3* carriers (*n* = 27,263) to *APOE3/4* carriers (*n* = 9,884) to *APOE4/4* carriers (*n* = 1,210), reflecting the escalating impact of *APOE4* allele dosage on AD risk (Extended Data Fig. 7). Strikingly, within each genotype group, extreme astrocyte aging consistently identified individuals at elevated AD risk compared to those with normal and youthful astrocyte aging, demonstrating that astrocyte aging provides independent risk stratification beyond *APOE* genotype.

To contextualize the prognostic power of astrocyte aging relative to established AD risk factors, we performed a comparative analysis against AD polygenic risk score (PRS), *APOE4* carrier status (present or absent) and chronological age (Fig. 3f). Excess AD risk associated with extreme astrocyte aging (HR = 5.16, 95% CI 4.06–6.56) and was comparable to *APOE4* carrier status (HR = 5.30, 95% CI 4.54–6.18), exceeding both PRS (HR = 2.14, 95% CI 1.92–2.39) and older chronological age (HR = 1.24, 95% CI 1.22–1.27). Individuals who carried the *APOE4* allele and had extreme astrocyte aging concurrently were at highest risk (HR = 11.58, 95% CI 8.56–15.66). Notably, sex-stratified analysis of AD risk revealed women were more vulnerable to the harmful associations linked to not only *APOE4* (HR = 5.82, 95% CI 4.73–7.16 for females compared to HR = 4.68, 95% CI 3.71–5.90 for males), but also astrocyte extreme aging (HR = 6.84, 95% CI 5.09–9.20 for females compared to HR = 3.54, 95% CI 2.35–5.34 for males). Possessing both *APOE4* and astrocyte extreme aging conferred a greater increase in AD risk for women (HR = 14.23, 95% CI 9.86–20.54) than men, further contextualizing sex-specific patterns of AD pathogenesis^40^. Most interestingly, youthful astrocytes reduced AD risk by over 60%.

Together, these findings highlight astrocyte aging as a potentially powerful biomarker that stratifies AD risk independently of and synergistically with *APOE* genotype. Maintaining youthful astrocyte function may be a potential therapeutic strategy to mitigate disease burden, especially in genetically predisposed individuals.

**Extended Data Fig. 8:**
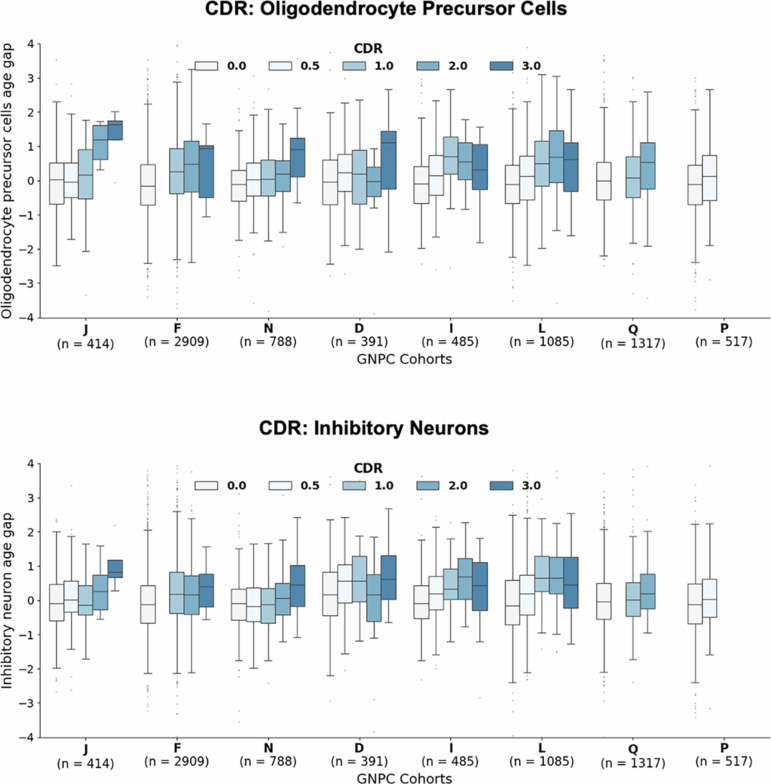
Box plot visualization of age gaps in oligodendrocyte precursor cells and inhibitory neurons stratified by Clinical Dementia Rating (CDR) scores across GNPC subcohorts. Box plots display the distribution of cell-type age gaps across CDR strata within each cohort (J, n = 414; F, n = 2,909; N, n = 788; D, n = 391; I, n = 485; L, n = 1,085; Q, n = 1,317; P, n = 517). In each box plot, the center line represents the median, the box bounds indicate the interquartile range (25th–75th percentiles), and whiskers extend to 1.5x the interquartile range, with points beyond this range represented as outliers.

Given the significant associations between cell type-specific aging and neurodegeneration, we further examined the relationship between cell-type age gaps and Clinical Dementia Rating (CDR) scores, a composite measure of dementia severity and general cognitive and functional performance^41^. Among all cell types, aging of oligodendrocyte precursor cells and inhibitory neurons demonstrated the strongest correlation with CDR (Extended Data Fig. 8). When visualizing age gaps across cohorts and stratifying by CDR score in the GNPC cohort, oligodendrocyte precursor cell aging showed a consistent stepwise increase with worsening cognitive impairment, with pronounced effects in cohorts J, F and N. Similarly, inhibitory neuron age gaps increased with higher CDR scores across cohorts. These observations further illustrate the biological relevance of cell type-specific aging in cognitive decline.

Furthering our analysis of cellular aging and AD, we examined age gap associations among participants in the NSHD cohort Insight-46 neuroimaging substudy (*n* = 483)^42^. We performed regression analyses evaluating independent associations between plasma pTau-217 burden (pg ml^−1^) and the Preclinical Alzheimer Cognitive Composite (PACC) score with cellular age gaps (Fig. 3h). Given plasma pTau-217 reliably identifies brain amyloid-β and tau-pathology with accuracy comparable to cerebrospinal fluid and PET biomarkers, it serves as a robust orthogonal measure for validating associations between cell type-specific aging and AD pathology^43^. Moreover, the PACC score is a validated measure sensitive to early cognitive decline that is widely used in preclinical dementia and AD research^44^.

Concordant with results obtained in the GNPC, we found significant associations between plasma phosphorylated tau-217 (pTau-217) burden and several neuronal and glial cell types, including astrocytes (coefficient beta (*ꞵ*) = 1.08, adjusted *P* = 3.92 × 10^−7^), oligodendrocyte precursor cells (*ꞵ* = 1.02, adjusted *P* = 1.19 × 10^−6^), oligodendrocytes (*ꞵ* = 0.76, adjusted *P* = 2.2 × 10^−4^) and inhibitory neurons (*ꞵ* = 0.76, adjusted *P* = 2.2 × 10^−4^) (Fig. 3h). For PACC, we observed an association between worsened cognition and elevated age gaps across a variety of cell types, with oligodendrocyte precursor cells demonstrating one of the strongest associations.

Collectively, these findings demonstrate that cell-type aging signatures derived from plasma proteomics are associated with neurodegenerative diseases across multiple cohorts and may offer granular insights into underlying pathophysiology at the cellular level.

We next investigated whether cellular aging signatures have prognostic value for cancer, stroke and chronic disease in the UKB cohort over 15 years of follow-up. Our findings reveal cellular aging signatures strongly prognosticate future disease.

**Fig. 4:**
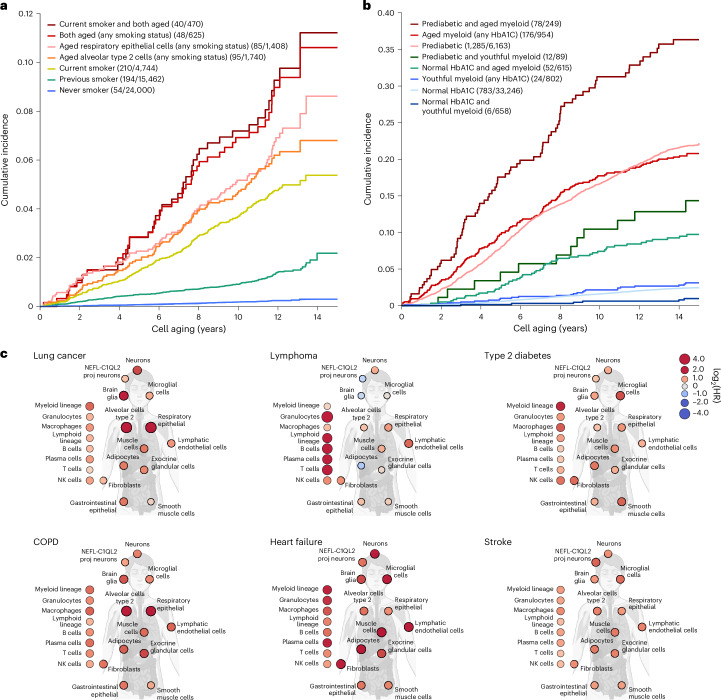
Cellular aging signatures predict cancer and chronic disease risk. **a**, Lung cancer cumulative incidence by smoking status and respiratory cell aging over 15 years of follow-up in the UKB cohort. Groups (highest to lowest incidence): current smokers with extreme aging in both alveolar type 2 and the broader respiratory epithelial lineage (*n* = 470); individuals with concurrent extreme aging (*n* = 625); respiratory epithelial lineage extreme aging (*n* = 1,408); alveolar type 2 cell extreme aging (*n* = 1,740); current smokers (*n* = 4,744); previous smokers (*n* = 15,462) and never smokers (*n* = 24,000). Case counts are indicated in the figure legend. **b**, Type 2 diabetes cumulative incidence by HbA1c status and myeloid lineage aging over 15 years of follow-up in the UKB cohort. Groups (highest to lowest incidence): prediabetic individuals with extreme myeloid lineage aging (*n* = 249); individuals with extreme myeloid lineage aging (*n* = 954); prediabetic individuals (*n* = 6,163); prediabetic individuals with youthful myeloid lineage aging (*n* = 89); individuals with normal HbA1c and extreme myeloid lineage aging (*n* = 615); individuals with youthful myeloid lineage aging (*n* = 658); individuals with normal HbA1c (*n* = 33,246); and individuals with normal HbA1c and youthful myeloid lineage aging (*n* = 648). Case counts are indicated in the figure legend. **c**, Body plots showing HRs for incident disease by extreme aged cell type, adjusted for chronological age and sex in the UKB cohort. Diseases shown are lung cancer, lymphoma, type 2 diabetes, COPD, heart failure and stroke. Displayed cell types represent the union of the top five prognostic cell types for each disease. Dot color and size represent log-transformed HR (log_2_(HR)). Illustrations in **c** created in BioRender; Augustina, V. https://biorender.com/7k7rct4 (2026).

For lung cancer, concurrent extreme aging in alveolar type 2 cells and the broader respiratory epithelial lineage was most prognostic (HR = 8.39, 95% CI 6.68–10.52; HR = 8.47, 95% CI 6.69–10.71, respectively) (Fig. 4a,c). Notably, in our study, these cellular aging signatures enhanced lung cancer risk stratification beyond the known risk factor of smoking status: current smokers with extreme aging in both cell types exhibited the highest risk (HR = 15.33, 95% CI 11.02–21.31), yielding 58% higher hazard than current smoking alone (HR = 9.69, 95% CI 8.04–11.68), while never smokers showed the lowest risk.

**Extended Data Fig. 9:**
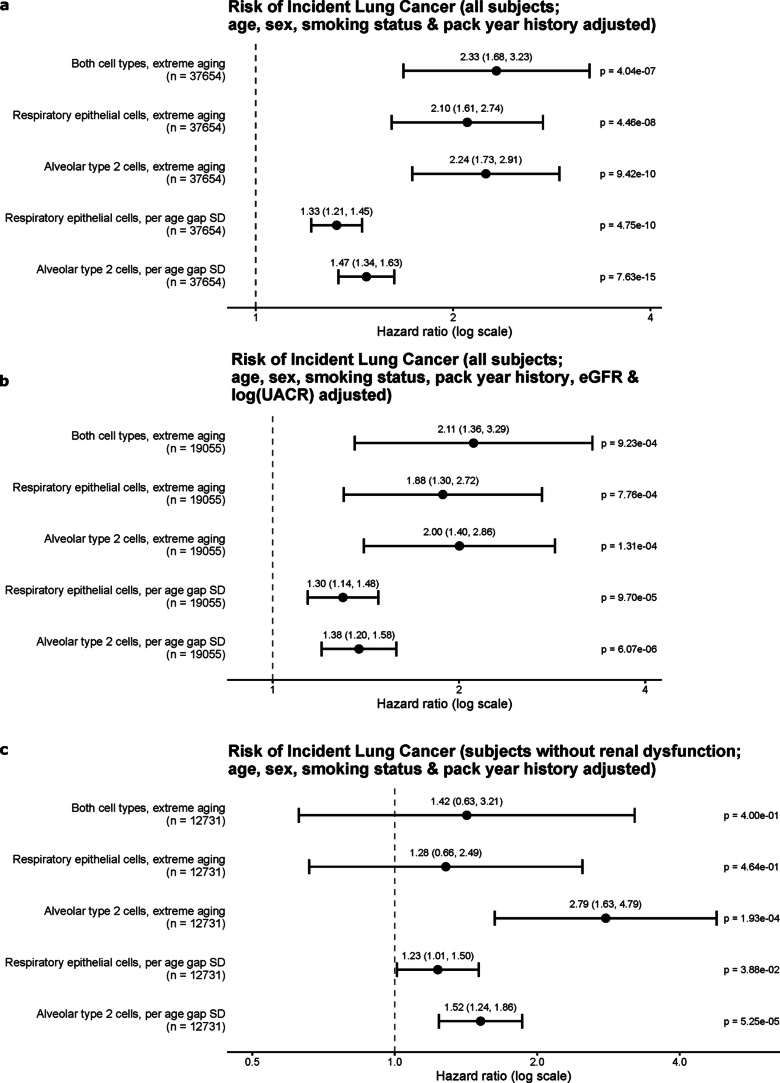
Alveolar type 2 and respiratory epithelial lineage aging have prognostic power for incident lung cancer. **(a)** Forest plot showing hazard ratios corresponding to the association between incident lung cancer and both alveolar type 2 cell and respiratory epithelial cell aging, evaluated with respect to both extreme aging status and continuous age gaps (z-scored). **(b)** HRs with additional adjustment for estimated glomerular filtration rate (eGFR) and log-transformed urine albumin-to-creatinine ratio (UACR). **(c)** Hazard ratios in a subpopulation restricted to individuals without evidence of kidney dysfunction (eGFR ≥ 90 mL/min/1.73m^2^ and normal urine albumin-creatinine ratio (A1); no adjustment for renal function variables. Statistical power limited by sample size: 289 subjects had extreme respiratory epithelial cell aging and no evidence of renal dysfunction; 124 subjects had extreme aging in both cell types and no evidence of renal dysfunction. All error bars indicate 95% CIs for Cox proportional hazards models, with baseline adjustment for chronological age and sex.

Notably, alveolar type 2 cell and respiratory epithelial lineage aging signatures retained independent prognostic value for incident lung cancer after adjustment for age, sex, smoking status and estimated pack-years (Extended Data Fig. 9). The pronounced prognostic value of alveolar type 2 cells, which serve as stem cells for repair and regeneration of the alveolus, is consistent with alveolar type 2 cells as the cell of origin for lung adenocarcinoma, the most common form of lung cancer. These findings align with literature suggesting that compromised regenerative capacity in the lung parenchyma may create a permissive environment for malignant transformation^45,46^.

For type 2 diabetes (Fig. 4b,c), myeloid lineage extreme aging demonstrated the strongest prognostic value (HR = 3.88, 95% CI 3.33–4.52) and remained significant after adjustment for established risk factors (Supplementary Fig. 8), consistent with a role of myeloid-derived cytokines in the initiation of an inflamed pancreatic islet microenvironment and increased susceptibility to type 2 diabetes^47^.

**Extended Data Fig. 10:**
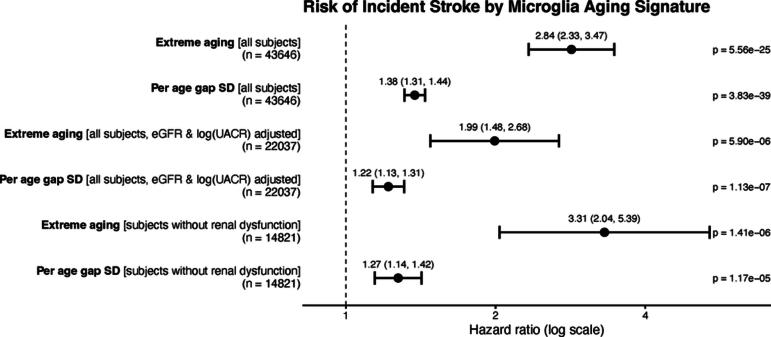
Microglia aging has prognostic power for incident stroke. Cox proportional hazard models were used to estimate hazard ratios with 95% CIs for incident stroke. Models evaluated microglia aging using both extreme aging status and continuous age gaps (z-scored). Sensitivity analyses included additional adjustment for renal function variables, including estimated glomerular filtration rate (eGFR) and log-transformed urine albumin-to-creatine ratio [log(UACR)]—or restriction to individuals without evidence of renal dysfunction (defined as eGFR 90 mL/min/1.73m^2^ and normal A1 albuminuria with UACR < 30 mg/g). All models had baseline adjustment for chronological age and sex.

For incident chronic obstructive pulmonary disease (COPD) (Fig. 4c), concurrent extreme aging in alveolar type 2 cells and the broader respiratory epithelial lineage showed the most prognostic power (HR = 6.31, 95% CI 5.57–7.13; HR = 5.45, 95% CI 4.75–6.25, respectively). For incident heart failure (Fig. 4c), extreme aging in muscle cells and fibroblasts was most prognostic (HR = 4.65, 95% CI 4.00–5.41; HR = 4.62, 95% CI 3.98–5.39, respectively), consistent with known mechanisms of dysfunctional remodeling following myocardial damage or with aging^48^. For incident stroke (Fig. 4c), extreme aging in NEFL-C1QL2 projection neurons demonstrated the strongest prognostic value (HR = 3.03, 95% CI 2.47–3.72), followed by microglia (HR = 2.84, 95% CI 2.33–3.47) (Extended Data Fig. 10).

Interestingly, for incident lymphoma (Fig. 4c), extreme aging in B cells was highly prognostic (HR = 6.63, 95% CI 4.25–10.33), following granulocytes (HR = 10.09, 95% CI 6.86–14.83) and T cells (HR = 6.75, 95% CI 4.33–10.54). In B cell lymphoma, dysregulation of both T-lymphoid and myeloid precursor-derived cells is known to contribute to malignancy pathogenesis^49,50^. This finding raises the question of whether coordinated aging across the hematopoietic niche may facilitate early lymphomagenesis. While difficult to compare directly, these HRs are higher than those for lymphoid clonal hematopoiesis of indeterminate potential and rival those of lymphoid mosaic chromosomal alterations^51^.

**Fig. 5:**
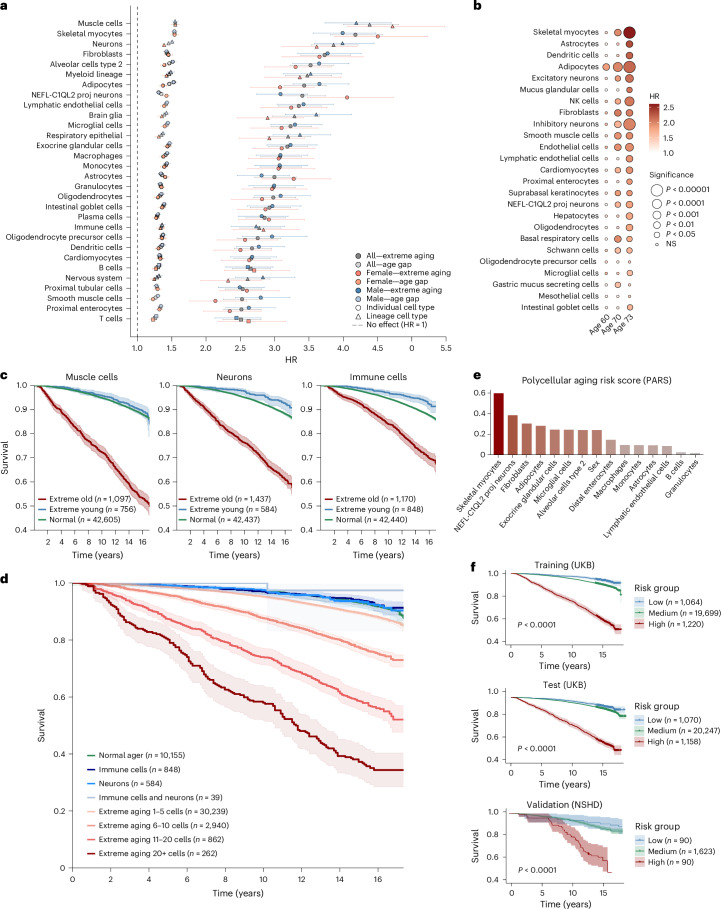
Cellular aging states inform survival and resilience. **a**, Analysis of cellular aging and all-cause mortality risk over 15 years of follow-up in the UKB cohort. HRs with 95% CI for all-cause mortality are shown, estimated using Cox proportional hazards models adjusted for chronological age and sex. Results shown for overall analysis (men and women combined, *n* = 44,458) and sex-stratified analyses (females: *n* = 24,046, males: *n* = 20,412). For each cell type, dark shaded markers represent estimates derived from extreme cellular aging and light shaded markers represent estimates derived from per-unit increase in age gap per cell type and or lineage. HRs correspond to exponentiated model coefficients, and horizontal lines denote 95% CIs. The top 30 cell types with the strongest associations with mortality risk are shown. **b**, HRs for all-cause mortality by cell type at three timepoints in the NSHD cohort (*n* = 364; baseline 63.2 ± 1.1 years, timepoint 2: 70.7 ± 0.7 years, timepoint 3: 72.9 ± 0.6 years; followed to a mean age of 78.2 ± 0.3 years). Associations between cell-type age gaps and mortality risk were assessed using Cox proportional hazards models, with time since blood draw as the time scale and death as the event. Models included *z*-scored age gaps with adjustment for chronological age and sex. Effect sizes are reported as HRs with 95% CIs. Two-sided *P* values were derived from Wald tests and adjusted using the Benjamini–Hochberg procedure. The top 25 cell types with strongest associations with mortality risk at the third timepoint are shown. **c**, Kaplan–Meier survival curves stratified by cellular aging status for muscle lineage (left), neuron lineage (middle) and immune lineage cell types (right) in the UKB cohort. Participants were categorized as extreme old (red; muscle: *n* = 1,097, neurons: *n* = 1,437, immune: *n* = 1,170), normal (green; muscle: *n* = 42,605, neurons: *n* = 42,437, immune: *n* = 42,440) or extreme young (blue; muscle: *n* = 765, neurons: *n* = 584, immune: *n* = 848) based on *z*-scored lineage and cell type age gaps. Lines represent Kaplan–Meier estimates of survival, and shaded bands indicate 95% CIs. Group differences were assessed using two-sided log-rank tests: muscle lineage, *P* < 0.0001; neuron lineage, *P* < 0.0001; immune lineage, *P* < 0.0001. **d**, All-cause mortality by number of extreme aging cell types over 15 years of follow-up in the UKB cohort. Kaplan–Meier curves stratified by extreme aging burden: normal agers (*n* = 10,155, ~90% survival); 1–5 extreme cell types (*n* = 30,239, ~85%); 6–10 (*n* = 2,940, ~73%); 11–20 (*n* = 862, ~52%); 20+ (*n* = 262, ~34%). Additional curves show individuals with youthful immune lineage (*n* = 848) or neuron lineage cell types (*n* = 584) irrespective of other cell-type aging patterns. Lines represent Kaplan–Meier estimates of survival, and shaded bands indicate 95% CIs. **e**, Polycellular aging risk score (PARS) for mortality stratification across plasma proteomics platforms and independent cohorts. Bar plot shows the top 15 cell-type contributors (model coefficients) for the PARS score. The PARS was derived from a multivariate Cox proportional hazards model trained in the UKB training cohort (*n* = 21,983). Predictors included binary extreme aging indicators and sex, with model selection performed using 10-fold cross-validation. Positive coefficients denote elevated mortality risk. **f**, Kaplan–Meier survival curves show risk stratification based on PARS in the UKB training cohort (top) (*n* = 21,983), UKB test cohort (middle) (*n* = 22,475) and NSHD validation cohort (bottom) (*n* = 1,803). Participants were categorized into low (bottom 5%), medium (middle 90%) and high (top 5%) risk groups based on cohort-specific score distributions. Lines represent Kaplan–Meier estimates of survival, and shaded bands indicate 95% CIs. Group differences were assessed using two-sided log-rank tests; with sample sizes and *P* values indicated in the figure legends.

Beyond disease prediction, we investigated whether cellular aging signatures have prognostic value for all-cause mortality in the UKB cohort using Cox proportional hazards models (Fig. 5a). The strongest associations were observed for extreme aging in muscle lineage cells (HR = 4.38, 95% CI 4.00–4.80) and skeletal myocytes (HR = 4.18, 95% CI 3.82–4.57), followed by neurons, fibroblasts, alveolar type 2 cells and myeloid lineage cells, with modest sex differences observed across cell types and lineages. These associations remained consistent after additional adjustment for renal function (Supplementary Figs. 9 and 10). When examining survival stratification by cellular aging status, extreme agers showed reduced survival compared to normal agers across all cell types (Supplementary Fig. 11). Representative survival curves for muscle lineage cells, neuron lineage cells and immune lineage cells are shown in Fig. 5c.

To investigate the temporal dynamics between cellular aging and mortality risk, we assessed associations between cellular aging and all-cause mortality across three longitudinal timepoints in the NSHD cohort (Fig. 5b). Tracking 364 individuals over a 15-year period (to mean age 78.2 $\pm$0.3 years), we found the prognostic value of cellular aging strengthened over time. This effect was most pronounced for skeletal myocytes, inhibitory neurons and astrocytes, which showed progressively increasing HRs across timepoints. By the third timepoint, over half of cell types were significantly associated with mortality (adjusted *P* < 0.05), indicating accumulation and convergence of aging signals.

We next examined how the cumulative burden of cellular aging influences survival by stratifying individuals according to their number of extreme aged cell types (Fig. 5d). A striking dose-response relationship emerged: individuals with normal aging profiles maintained ~90% survival, whereas those with over 20 extremely aged cell types showed ~34% survival over a 15-year follow-up period. Intermediate groups showed graded risks. Individuals with youthful immune lineage or neuron lineage cell types showed survival comparable or better than normal agers, with combined youthful states showing the highest survival.

The observation that mortality risk depends on both specific cellular vulnerabilities and cumulative aging burden motivated us to develop an integrated polycellular aging risk score (PARS) encompassing a composite of cell types (Fig. 5e). The PARS model was trained (*n* = 21,983) and tested (*n* = 22,475) in the UKB cohort (Olink) and validated in the NSHD cohort (*n* = 1,803; SomaScan). Individuals were stratified into high (top 5%), low (bottom 5%) and medium (all others) mortality risk groups using cohort-specific score distributions. Kaplan–Meier analyses showed clear risk stratification in training and test sets (log-rank *P* < 0.0001), with reduced survival among high-risk individuals (Fig. 5f). This finding was reproduced in the validation cohort despite differences in proteomics assay technology. Consistent with its prominence as a predictor of mortality, skeletal myocyte aging exhibited the largest coefficient in the PARS model^52^. These findings illustrate the prognostic value of the PARS as a platform-agnostic biomarker for mortality and health span.

## Discussion

Our study introduces a cell type-specific framework for modeling biological aging with plasma proteomics, leveraging the measurement of over 7,000 plasma proteins across more than 60,000 individuals in three independent cohorts.

By deriving cell type-specific aging models and age gaps, we found both substantial heterogeneity and coordinated shifts in biological aging at the cellular level, including varied onset of extreme aging that could reflect distinct disease vulnerabilities. Our framework uncovered significant associations between cellular aging and neurodegenerative diseases, with prognostic value years before clinical onset. Notably, extreme skeletal myocyte aging demonstrated pronounced prognostic power for incident ALS over 15 years of follow-up in the UKB cohort, even for cases diagnosed more than 3 years after baseline assessment, suggesting potential for presymptomatic detection.

For AD, analysis in the GNPC revealed accelerated aging across multiple cell types, underscoring the systemic nature of the disease^53^. These associations were further validated in the NSHD cohort and supported by links to cognitive phenotypes. Notably, oligodendrocyte precursor cell aging was consistently associated with worsened cognition across cohorts, as measured by CDR and PACC. These findings motivate further investigation into the potential role of impaired oligodendrocyte lineage regeneration and myelin maintenance in age-related cognitive decline^54^.

Astrocyte aging showed the strongest association with incident AD in the UKB cohort, comparable to *APOE4* carrier status and exceeding polygenic risk score and chronological age. Of interest, astrocyte aging interacted with *APOE* genotype, with *APOE4* homozygotes exhibiting 3-fold higher cumulative incidence of AD for extreme versus normal astrocyte aging over a 15-year follow-up period. The risk gradient persisted across genotypes, with women identified as more vulnerable to these effects than men. Youthful astrocytes seem to powerfully attenuate the detrimental effects of *APOE4*. Pending replication, this interaction may point to mechanisms of brain resilience.

Cellular aging signatures also had prognostic value for incident cancer and chronic diseases. Extreme respiratory cell aging identified smokers at elevated lung cancer risk while myeloid lineage aging identified normoglycemic individuals at higher type 2 diabetes risk, underscoring potential for targeted surveillance and early preventive interventions^55^.

Our findings suggest that specific cellular vulnerabilities and cumulative aging burden influence survival. Skeletal myocyte aging showed the strongest association with mortality, followed by neurons, fibroblasts, alveolar type 2 cells and myeloid lineage cells; implicating musculoskeletal, cognitive, pulmonary and immune maintenance in longevity^56^. A striking dose-response relationship revealed that individuals with extreme aging across 20+ cell types had ~34% survival over 15 years of follow-up compared to ~90% for those posessing normal aging profiles, with graded risks for intermediate groups. Youthful immune lineage and neuron lineage cell types attenuated mortality risk. Furthermore, our findings reveal a composite cell type polycellular aging risk score (PARS) model maintained robust mortality risk stratification across cohorts and proteomic platforms, demonstrating its potential as a platform-agnostic biomarker for healthspan.

Altogether, the noninvasive blood-based approach presented in this study enables biological aging to be characterized at cellular resolution, providing a new framework for studying aging heterogeneity and potential therapeutically relevant disease mechanisms. Future work should explore the sequential progression and genetic basis of cellular aging. Moreover, a complimentary analysis of lifestyle factors and superager cohorts could identify proteomic signatures underlying exceptional longevity, resilience and rejuvenation at the cellular level.

Our study has limitations. Analysis was based on and is thus restricted to cell types cataloged in the Human Protein Atlas, leaving certain specialized cell populations underrepresented. Transcript levels do not always reflect protein abundance, and some cell-type assigned proteins such as cardiomyocyte-assigned FABP3 and microglia-assigned HAVCR1 may be produced or released more broadly under specific circumstances. Accordingly, plasma proteins arise from multiple cellular processes not explicitly disentangled here^57^. Due to the fact cohorts were predominantly older and Caucasian, broader validation in younger and more diverse populations is essential to extend the generalizability of results reported in this study.

In summary, large-scale plasma proteomics combined with machine learning enables noninvasive assessment of cell type-specific aging in living populations and reveals strong links between cellular aging, disease and mortality.

## Methods

### Proteomics measurements

#### SomaScan proteomics

The SomaLogic (https://SomaLogic.com) SomaScan assay, which uses slow off-rate modified DNA aptamers (SOMAmers) to bind target proteins with high specificity, was used to quantify plasma protein abundance in the GNPC, KADRC and NSHD cohorts. The v4.1 assay (7,289 proteins) was used for GNPC and KADRC, whereas the NSHD cohort used the v5.0 assay (11,742 proteins). Standard SomaLogic normalization, calibration and quality control were applied, yielding protein measurements in relative fluorescence units. Pooled reference and buffer standards were included on each plate to control for batch effects, and samples were normalized within and across plates using median reference signals. Additional normalization to a pooled reference was performed using an adaptive maximum likelihood procedure. The resulting values, provided by SomaLogic, were treated as raw data, with downstream preprocessing performed by GNPC and contributing cohorts. To enable application of cellular aging clocks trained on v4.1 proteins in NSHD, v5.0-to-v4.1 scaling factors provided by SomaLogic were applied to raw abundance values before age estimation.

#### Olink proteomics

The Olink Explore 3072 proximity extension assay platform was used to quantify plasma proteins in the UKB cohort. Olink proteomics is based on the binding of two polyclonal antibody pools to a target protein and subsequent hybridization and enrichment of two unique single-stranded DNA probes. The assay encompasses 2,941 immunoassays targeting 2,923 proteins. Olink measurements were based on normalized protein expression (NPX) values recommended by the manufacturer, which included normalization. Additional details on Olink proteomics data quality control, acquisition and handling in the UKB cohort are well-documented^58^ (further details are available at https://biobank.ndph.ox.ac.uk/ukb/ukb/docs/PPP_Phase_1_QC_dataset_companion_doc.pdf).

### Study population and clinical phenotypes

#### Global Neurodegeneration Proteomics Consortium

The GNPC v1.3 dataset (https://www.neuroproteome.org/new-harmonized-data-set) is a large-scale global neurodegenerative disease biomarker discovery effort that hosts over 40,000 patient samples from over 20 international research groups. Data from the GNPC include biofluid samples from clinically healthy individuals, and those diagnosed with AD, PD, ALS, MCI–SCI and FTD. All cohorts and data were anonymized with letter codes by GNPC before analysis. A standard sample collection procedure was utilized, with potential site-to-site variation guided by the discretion of individual cohorts. Ethics approval for each cohort was obtained from the respective Institutional Review Boards, and written informed consent was obtained from all participants or their legally authorized representatives.

Our initial analysis included 21,979 individuals in GNPC and was further reduced to account for reporting completeness and consistency. Specifically, we retained v4.1 (7,289 protein targets) SomaScan assay data and only included individuals with complete age and sex information. Due to known variations in sample quality and detection capabilities in serum and CSF, we limited our analysis to plasma samples collected in ethylenediaminetetraacetic acid (EDTA) by routine venipuncture. From this, 14,281 individuals from 14 independent cohorts were prioritized for downstream analysis (Supplementary Table 3). Among these individuals, 7,074 were defined as healthy. In agreement with the definition proposed by GNPC, we define the healthy population as individuals with no diagnosis of AD, PD, FTD, MCI or ALS, and possessing a CDR score not exceeding zero.

#### Knight Alzheimer’s Disease Research Center

The KADRC cohort is a National Institute on Aging-funded longitudinal observational study of clinical dementia participants and age-matched controls. Research participants within KADRC undergo longitudinal cognitive, neuropsychological, imaging and biomarker assessments including CDR testing. The KADRC cohort includes samples from both individuals with AD diagnosis and healthy controls. Blood collection and processing were performed in accordance with prior-reported rigorous standardized protocols to minimize variation associated with blood draw and processing. All blood draws were performed in the morning to minimize the impact of circadian rhythm on protein concentrations. The Institutional Review Board of Washington University School of Medicine in St Louis approved the study (Institutional Review Board number 201109148), and research was performed in accordance with the approved protocols. Written informed consent was obtained from all participants or their family members.

#### National Survey of Health and Development

The NSHD is a longitudinal birth cohort of individuals born in a single week in March 1946 in the United Kingdom. Plasma proteomics were measured at three timepoints (mean ages ± s.d.: 63.2 ± 1.1 years, *n* = 1,803; 70.7 ± 0.7 years, *n* = 483; and 72.9 ± 0.6 years, *n* = 364), with 364 participants tracked across all three timepoints. Survival data were obtained from National Health Service (June 2024) via the National Health Service England and the National Health Service Central Register, with follow-up to a mean age of 78.2 ± 0.3 years. Individuals who withdrew were censored (*n* = 2). A subset of participants (*n* = 483) were included in the Insight-46 neuroimaging substudy. In this subgroup, plasma pTau-217 was quantified using the ALZpath Simoa assay (Quanterix). Cognitive function was assessed using the PACC, a composite measure sensitive to early cognitive decline. The NSHD study was approved by the National Research Ethics Service Committee (14/LO/1173). All participants provided written consent.

#### UK Biobank

The UKB is a population-based prospective observational cohort with omics and phenotypic data collected from approximately 500,000 participants, aged 40 to 69 years, spanning a recruitment period between the years 2006 and 2010. Participants underwent baseline assessments including physical measurements, questionnaires and biological sample collection, with longitudinal follow-up for disease outcomes and mortality extending over 15 years. All participants provided informed consent.

The UKB Pharma Proteomics Project generated Olink Explore 3072 plasma proteomics data for ~54,000 participants at their baseline visit. After UKB Pharma Proteomics Project quality control, 2,923 proteins were measured in ~53,000 samples. Additional filtering removed samples with >1,000 missing proteins (*n* = 8,182), discordant reported and genetic sex (*n* = 373) and proteins missing in >20% of samples (*n* = 7), yielding a final dataset of 44,458 samples and 2,916 proteins. Data were split into training (*n* = 21,983) and test (*n* = 22,475) sets by assessment center, with protein values *z*-score normalized using training set parameters. Missing values (2.7%) were imputed using *k*-nearest neighbors (*k* = 148; sklearn KNNImputer v1.3.1), with performance validated by masked-value reconstruction (mean absolute error = 0.57).

Disease diagnosis dates were collated from UKB ‘First Occurrences’ (data category 1712), ‘Cancer Register’ (data category 100092), and, where available, ‘algorithmically defined outcomes’ (data category 47; for example for AD, PD, ALS, FTD and stroke). If multiple dates were recorded, the earliest date was used. The following ‘First Occurrences’ or ‘Cancer Register’ ICD-10 (and where available ICD-9) codes were used: heart failure (I42, I43 and I50); stroke (I60–64); COPD (J41–44); type 2 diabetes (E11 and E14); lymphoma (C81–89, and ICD-9 2014–2016, 2019 and 2020); lung cancer (C33, C34 and ICD-9 1623 and 1629); AD (F00 and G30) and PD (G20). For calculation of type 2 diabetes incidence, all individuals with diagnosis of type 1 diabetes (ICD-10 E10) or other uncommon forms of diabetes (‘other specified diabetes’ ICD-10 E13) were removed from analysis. Age at diagnosis was calculated by comparing the month and year of birth to date of diagnosis. Time to diagnosis was found by comparing age at diagnosis to calculated or reported age at assessment.

Type 2 diabetes diagnosis date was derived from the earliest diagnosis date from the ‘First Occurrences’ category or first date outpatient hemoglobin A1c value was greater than or equal to 6.5%. Individuals diagnosed with dementia without specified cause and without evidence of alternative etiology (neurodegenerative disease of the basal ganglia, ALS, multiple sclerosis, syphilis, HIV, B12/folate/thiamine deficiency, frontotemporal dementia, hydrocephalus, alcohol use disorder (either diagnosed or meeting criteria via reported consumption: greater than 28 units per week for women or 35 units per week for men), cerebrovascular pathology, sedative abuse, ICD-10 A81 ‘atypical virus infections of central nervous system’ or ICD-10 G32 ‘other degenerative disorders of nervous system’) were assigned a diagnosis of AD given its status as the predominant cause of dementia. Individuals with competing diagnoses (for example, FTD and AD or progressive supranuclear palsy and PD) were classified as having only the rarer diagnosis given the high clinical index of suspicion needed for formal diagnosis. Individuals with nonphysiologic diagnosis dates were removed from disease incidence analysis of the corresponding disease.

The following variables were extracted from UKB baseline assessment: smoking status (data field 20116_i0), pack-years of smoking (data field 20161_i0), hemoglobin A1c (data field 30750_i0), BMI (data field 21001_i0), waist circumference (data field p48_i0), alcohol consumption (aggregated over data fields 1568_i0, 1578_i0, 1588_i0, 1598_i0 and 1608_i0), sleep duration (data field 1160_i0), physical activity (data field 884_i0 and 904_i0), plasma creatinine (data field 23478_i0), plasma cystatin C (data field 30720_i0), urine creatinine (data field 30510_i0), urine albumin (data field 30600_i0), *APOE* genotype (data field 2315) and AD PRS (data field 26206). Individuals not reported as having any *APOE2* or *APOE4* allele were assigned *APOE3/3* genotype. Healthy lifestyle was defined as meeting the following: never smoking, no regular alcohol consumption, BMI < 25, waist circumference <90 cm for men or <84 cm for women, at least 5 days per week of 10+ min of moderate or vigorous physical activity and ≥7 h of sleep per night. Details on available phenotypes can be found at https://biobank.ndph.ox.ac.uk/showcase/.

### Identification of cell type-enriched plasma proteins

The Human Protein Atlas single-cell RNA-seq database (Human Protein Atlas version 24.1 www.proteinatlas.org/humanproteome) was used to map the putative cell type-specific plasma proteome. The single-cell transcriptomics dataset utilized in this study encompassed more than 60 cell types and over 20,000 genes (normalized transcripts per million; a description of all included cell types is provided in Extended Data Fig. 1). Hofbauer, Langerhans and Kupffer cells were pooled into a broader macrophage categorization. Sex-specific cell types were excluded from the study. Guided by prior studies, we classified genes as ‘cell-type-enriched’ if they were expressed at least twofold higher in one cell type compared to any other cell type or expressed exclusively in a single cell type. Cell-type-enriched genes were mapped to the 7,289 plasma proteins quantified in the v4.1 SomaScan assay for SomaScan aging clock models, and to the 2,923 plasma proteins quantified in the Olink Explore 3072 assay for Olink aging clock models. Of note, while gene expression in tissues does not guarantee secretion into plasma, studies have shown that a substantial proportion of cell-type-enriched transcripts correspond to detectable proteins in blood^57^.

### Estimation of cellular plasma protein trajectories

Plasma protein expression was *z*-scored (that is standardized to mean = 0, s.d. = 1) and modeled as a function of chronological age using LOWESS regression (frac = 0.3; statsmodels v0.14.4). Resulting trajectories were used for heatmap visualization and clustering (scipy.cluster.hierarchy v1.15.1). Pairwise Euclidean distances were computed and hierarchical clustering was performed using Ward’s method. The optimal number of clusters was determined using the elbow method, yielding 33 clusters (threshold = 0.05).

### Trajectory clustering with cell-type and pathway enrichment analysis

To identify cell types that were enriched in clusters, we performed hypergeometric enrichment analysis. For each cluster, we assessed the overlap between cluster proteins and predefined sets of proteins mapped to individual cell types. For each cell type, a hypergeometric test was used to compute the probability of observing the actual or greater overlap by chance, given the size of the cluster, the size of the mapped protein set and the size of the overall background. To account for multiple testing, false discovery rate correction was applied with the Benjamini–Hochberg procedure using the stats.multitest.multipletests function in the statsmodels (v0.14.4) python package. Cell types enriched in a cluster were defined as those meeting the significance threshold with adjusted *P* < 0.05. Cell types meeting this threshold were plotted in a dot plot. Organs were incorporated into this analysis using an identical method. Mapping of the organ-specific proteome was performed utilizing methods from prior studies and publicly available datasets provided by the Genotype Tissue Expression project.

Pathway enrichment analysis was performed for each cluster using GProfiler (gprofiler-official v1.0.0) with the human gene namespace (organism = ‘hsapiens’), with restriction to Gene Ontology, Biological Process, Molecular Function, Cellular Component, Kyoto Encyclopedia of Genes and Genomes and Reactome databases.

### Cellular age estimation and age gap calculation

To estimate biological age using plasma proteomics, we developed cell type-specific aging models using elastic net regression with the glmnet R package (v4.1.7). Each aging model was trained on a distinct set of cell-type-enriched plasma proteins that were used to predict chronological age. We implemented bootstrap aggregation, generating 100 bootstrap samples through resampling with replacement from our training data. For each bootstrap sample, we trained a model on *z*-scored log-transformed protein abundance values with sex (F = 1, M = 0) as an additional covariate to predict chronological age. Hyperparameter tuning of the L1 regularization parameter (λ) was performed using 10-fold cross-validation with the cv.glmnet function. The final predicted age for each sample was calculated as the mean prediction from the 100 bootstrap models. We applied this approach to 60 cell types, excluding 15 sex-specific cell types to ensure representation of both sexes in cell-type biological age estimation.

For the SomaScan platform, models were trained on healthy individuals in the KADRC cohort and subsequently applied to the broader GNPC cohort for disease association analyses, and to the independent NSHD cohort for external validation. Aging models were evaluated based on performance and robustness criteria. Models were retained if they exhibited sufficient predictive performance (correlation coefficient *r* ≥ 0.25 in training, *r* ≥ 0.15 in test; minimum 4 protein features). Including models with fewer than 4 features can limit the robustness of downstream inferences due to the inherent technical noise in proteomic assays. After applying these quality control criteria, 43 cellular aging models were retained for downstream analyses.

For the Olink platform, models were trained on 21,983 individuals from the UKB cohort training set and subsequently applied to the UKB cohort test set (*n* = 22,475). Since the Olink dataset included 2,916 proteins after quality control—fewer than the 7,289 proteins measured by SomaScan—and generally yields fewer signatures across cell types, we additionally developed 14 lineage-level cell-type models to capture aging signals at broader ontological categories. To demonstrate the capability of our framework to model cellular aging at multiple levels of the cell-type ontology, we extended our analysis to include 14 lineage cell types, including the below broad parental categories with associated subgroups:

#### Immune lineage group

Lymphoid lineage (B cells, plasma cells, T cells and NK cells); myeloid lineage (monocytes, granulocytes, macrophages and dendritic cells) and all immune cells (combining all lymphoid and myeloid cell types).

#### Nervous system lineage group

Neuron lineage (excitatory and inhibitory); brain glia lineage (astrocytes, oligodendrocytes, oligodendrocyte precursor cells and microglial cells); retinal cell lineage (cone photoreceptor, rod photoreceptor, bipolar, horizontal and Müller glia cells), and all nervous system (all neuronal and glial cell types including Schwann cells).

#### Endothelial lineage group

Vascular endothelial cells and lymphatic endothelial cells.

#### Glandular lineage group

Breast glandular cells, exocrine glandular cells, prostatic glandular cells, pancreatic endocrine cells, mucus glandular cells, serous glandular cells, salivary duct cells and secretory cells.

#### Epithelial lineage group

Respiratory epithelial lineage (alveolar type 1 cells, alveolar type 2 cells, basal respiratory cells, ciliated cells, club cells and ionocytes); skin epithelial lineage (basal keratinocytes, suprabasal keratinocytes, melanocytes, basal squamous epithelial cells and squamous epithelial cells); gastrointestinal epithelial lineage (distal enterocytes, proximal enterocytes, intestinal goblet cells, Paneth cells, enteroendocrine cells and gastric mucus-secreting cells), and urogenital epithelial lineage (collecting duct cells, distal tubular cells and proximal tubular cells).

#### Muscle lineage group

Skeletal myocytes, cardiomyocytes and smooth muscle cells.

For each lineage cell type, we identified protein signatures uniquely enriched within the lineage, defined as genes with average expression at least twofold higher than any cell type outside the lineage. This approach captures shared lineage-level aging signals while maintaining biological interpretability. After applying the same quality control criteria (correlation coefficient *r* ≥ 0.25 in training, *r* ≥ 0.15 in test; minimum 4 protein features), 48 cell type-specific aging models were retained for Olink-based analyses (including both individual cell types and lineages).

To calculate individual age gaps for each cell type-specific aging model, we fit a local regression between predicted and chronological age using the LOWESS function from the statsmodels (v0.14.4) python package with the fraction parameter set to 2/3 to estimate the population mean. We derived age gaps for each cohort based on the fit of healthy individuals in the corresponding cohorts to account for cohort differences and excluded two cohorts (out of 16) that lacked healthy individuals in GNPC. Individual sample age gaps were calculated as the residual between predicted age and the LOWESS regression estimate of the population mean for the corresponding chronological age. Age gaps were *z*-scored (that is standardized to mean = 0 and s.d. = 1) per aging model to account for differences in model variability and facilitate comparison across cell types in downstream analyses. For each cell type, we classified individuals based on ‘extreme’ deviation of biological from chronological age, defined as having an absolute *z*-scored age gap over 2 (that is, extreme agers >2, youthful agers <−2)

### Cellular aging correlation analyses and network visualization

We examined correlations of age gaps across cell types using Pearson correlation with the pearsonr function from the SciPy Python package (v1.15.1). In the network visualization, each node represents a specific cell type, colored according to its cellular category. Edges connect cell type pairs with age gap correlations exceeding a threshold of 0.35.

### Extreme aging stability analysis

The stability of extreme aging cellular states was evaluated in the NSHD cohort across 3 timepoints spanning a 10-year period (*n* = 364; mean ages and s.d.: 63.2 $\pm$1.1 years, 70.7 $\pm$0.7 years and 72.9 $\pm$0.6 years), wherein the retention of extreme agers from baseline to the final timepoint was quantified. Only individuals with proteomic samples at all 3 timepoints were included in the extreme aging stability analysis. Alluvial plots were constructed using Python packages pandas (v2.1.1), NumPy (1.24.4), and Matplotlib (v3.8.0).

### Association analysis between age gaps and neurodegenerative disease status

Associations between cell type-specific age gaps and neurodegenerative disease diagoses were evaluated using point-biserial correlation with the pointbiserialr function from SciPy (v1.11.3). The analysis included the 5 neurodegenerative conditions present in the GNPC: AD (*n* = 2,761), ALS (*n* = 245), PD (*n* = 476), FTD (*n* = 199) and MCI–SCI (*n* = 1,992). Only cohorts with at least 10 disease cases and healthy controls were included in the corresponding analyses. For each disease analysis, healthy individuals from the relevant cohorts containing the disease cases served as controls.

### Prognostic analysis of incident disease

The prognostic value of cellular aging signatures for incident disease in the UKB cohort was assessed using Cox proportional hazards models with 15 years of follow-up using the lifelines package in Python (v0.27.8) and R package survival (v3.8.3). Participants were followed until disease onset, death, and or last clinical update. HRs and 95% CIs were estimated for both continuous (*z*-scored age gaps) and extreme aging status. Models were adjusted for chronological age and sex, with additional covariates included for specific diseases or further analysis as specified (for example, HbA1c, BMI, smoking, pack-years and renal function for type 2 diabetes).

For AD, we compared the prognostic value of cellular aging signatures against established risk factors. HRs were computed for *APOE4* carrier status, *APOE2* carrier status, AD PRS, chronological age, astrocyte aging status and *APOE* genotype combined with astrocyte aging status. Sex-stratified analyses were performed by fitting separate Cox models for females and males to assess potential sex differences in AD risk associations. For ALS, a sensitivity analysis was performed considering only cases diagnosed more than 3 years after blood draw to assess whether the prognostic value was retained.

Cumulative incidence curves were estimated using the Kaplan–Meier method (lifelines v0.27.8) and visualized for key cell type-disease associations stratified by aging status. Differences in survival between groups were assessed using log-rank tests.

### All-cause mortality analysis

We evaluated the association between cellular aging signatures and all-cause mortality in the UKB cohort using Cox proportional hazards regression models adjusted for chronological age and sex (lifelines v0.27.8 or survival v3.4.2) over 15 years of follow-up. For each cell type, we computed HRs and 95% CIs for both extreme aging status and continuous age gaps. Sex-stratified analyses were performed by fitting separate Cox models for females and males, adjusting for chronological age. Results were visualized using forest plots, with separate estimates shown for overall, female and male populations.

Kaplan–Meier survival curves were generated to visualize mortality risk stratified by cellular aging status (extreme, normal and youthful) for key cell types. Differences in survival between groups were assessed using log-rank tests. To examine the cumulative burden of cellular aging on survival, we stratified individuals by the total number of cell types exhibiting extreme aging and generated Kaplan–Meier curves for each stratum. Cox proportional hazards regression, Kaplan–Meier estimation and log-rank tests were applied using lifelines (v0.27.8).

### Association of cellular aging with AD pathology and cognitive performance

We assessed whether cell types linked to AD diagnosis in GNPC were associated with biological evidence of AD pathology and cognitive performance in the NSHD cohort Insight-46 substudy (*n* = 483). Using AD-relevant measures available in the study, we applied linear regression models adjusted for sex and chronological age to evaluate the association between *z*-scored cellular age gaps and pTau-217 (pg ml^−1^) burden. Plasma pTau-217 was measured using the ALZpath assay on the Single Molecule Array (Simoa) HD-X platform. To assess cognitive function, we applied linear models to evaluate associations between cellular age gaps and the PACC score, with additional adjustment for childhood cognitive ability. Continuous variables were standardized (*z*-scored) before analysis, such that regression coefficients represent effect sizes in s.d. units. The analysis was applied to the 22 cell types linked to clinical AD in the GNPC cohort. Linear regression models were fitted using the stats package in R (v4.1.2).

### Extreme ager odds ratio analysis

We assessed the association between extreme aging in specific cell types and neurodegenerative disease diagnosis by calculating odds ratios (OR) using Fisher’s exact test. For each cell type-disease pair, we constructed contingency tables comparing the frequency of extreme agers (*z*-scored age gap greater than 2) versus non-extreme agers among disease cases versus healthy controls. Fisher’s exact test was performed using the fisher_exact function from the SciPy Python package (v1.15.1). The Haldane–Anscombe continuity correction was applied by adding one or two to each cell in the contingency tables to mitigate potential bias from zero counts. The 95% CIs for OR were computed using Woolf’s method.

### Longitudinal mortality risk analysis in the National Survey of Health and Development

We analyzed the NSHD cohort to compute sequential estimates of mortality risk over a 10-year period using age gap estimates of same chronological aged individuals at three timepoints (*n* = 364; mean ages 63.2 $\pm$1.1 years, 70.7 $\pm$0.7 years and 72.9 $\pm$0.6 years). Cox proportional hazards models were used to examine associations between cellular age estimates and mortality risk over ~15 years of follow-up, with censoring for withdrawal from follow-up (*n* = 2) or death (*n* = 281) and adjustment for chronological age and sex. The R package survival (v3.4.2) was used. For each cell type, we computed HRs and 95% CIs for continuous *z*-scored age gaps.

### Development and cross-platform validation of the polycellular aging risk score (PARS)

The PARS model was developed by modeling binary extreme aging status across all cell types using multivariate Cox regression in the UKB cohort training set (*n* = 21,983; Olink). The use of binary extreme aging features (present or absent) rather than continuous age gap values enhances robustness to platform-specific technical variation and facilitates cross-platform generalization. Model coefficients were used to compute individual PARS scores, and participants were stratified into high (top 5%), medium (middle 90%), and low (bottom 5%) risk groups based on cohort-specific score distributions. The PARS model was validated in the UKB test set (*n* = 22,475) and the independent NSHD cohort (*n* = 1,803; SomaScan) using the same percentile categorizations. Survival differences between risk groups were evaluated using Kaplan–Meier curves and log-rank tests (lifelines v0.27.8).

### Statistical analyses

Pearson correlation was implemented using the pearsonr function from the SciPy Python package (v1.15.1). Pairwise *t*-tests were performed to compare cellular age gaps between *APOE* genotype groups using the ttest_ind function from SciPy. Associations between cell type-specific age gaps and neurodegenerative diseases were evaluated using point-biserial correlation with the pointbiserialr function from SciPy (v1.15.1). FDR correction for multiple hypothesis testing was applied using the Benjamini–Hochberg procedure in all relevant statistical analyses with the multipletests function from statsmodels (v0.14.0), with a significance threshold of 0.05.

### Reporting summary

Further information on research design is available in the Nature Portfolio Reporting Summary linked to this article.

## Online content

Any methods, additional references, Nature Portfolio reporting summaries, source data, extended data, supplementary information, acknowledgements, peer review information; details of author contributions and competing interests; and statements of data and code availability are available at 10.1038/s41591-026-04446-y.

## Supplementary information

Supplementary Information Supplementary Figs. 1–11 and GNPC Consortium Author List. A list of individuals who are affiliated with the GNPC but were not direct authors of the manuscript is provided on the final page of the combined Supplementary Information.

Reporting Summary

Supplementary Tables 1–12 Supplementary Tables 1–12.

## Data Availability

GNPC data is available upon request to qualified researchers through a standard protocol (www.neuroproteome.org/harmonized-data-set-hds). Access is contingent on adherence to the GNPC Data Use Agreement and the Publication Policies. The Knight-ADRC proteomics data were generated by the laboratory of C.C. (cruchagac@wustl.edu) and can be requested at https://knightadrc.wustl.edu/professionals-clinicians/request-center-resources/submit-a-request. UKB data are available upon request through a standard protocol (www.ukbiobank.ac.uk/register-apply). Bona fide researchers can apply to access the NSHD data via a standard application procedure (further details available at https://skylark.ucl.ac.uk/NSHD/access/). Mortality data can be requested from the UK Longitudinal Linkage Collaboration (https://ukllc.ac.uk/). Access to controlled datasets requires submission of a formal application and data use agreement; requests are reviewed by the relevant data access committees and typically receive a response within approximately 4–12 weeks.

## Code availability

The code used to generate cell-type proteomic aging clocks and perform biological age estimation is publicly available at https://github.com/dingdaisy/cellage. This repository includes model training scripts, preprocessing code and instructions for reproducing key analyses.
