# Supplementary material for: Plasma proteomic signatures of cellular aging predict human disease
Source: Nat Med. 2026 Jun 15;32(6):2060–72. doi: 10.1038/s41591-026-04446-y (PMC13279268; doi:10.1038/s41591-026-04446-y)
Supplement: Supplementary file 1 — Supplementary Figs. 1–11 and GNPC Consortium Author List. A list of individuals who are affiliated with the GNPC but were not direct authors of the manuscript is provided on the final page of the combined Supplementary Information. [file 41591_2026_4446_MOESM1_ESM.pdf]

nature medicine

Supplementary information https://doi.org/10.1038/s41591-026-04446-y

Plasma proteomic signatures of cellular aging predict human disease

In the format provided by the authors and unedited

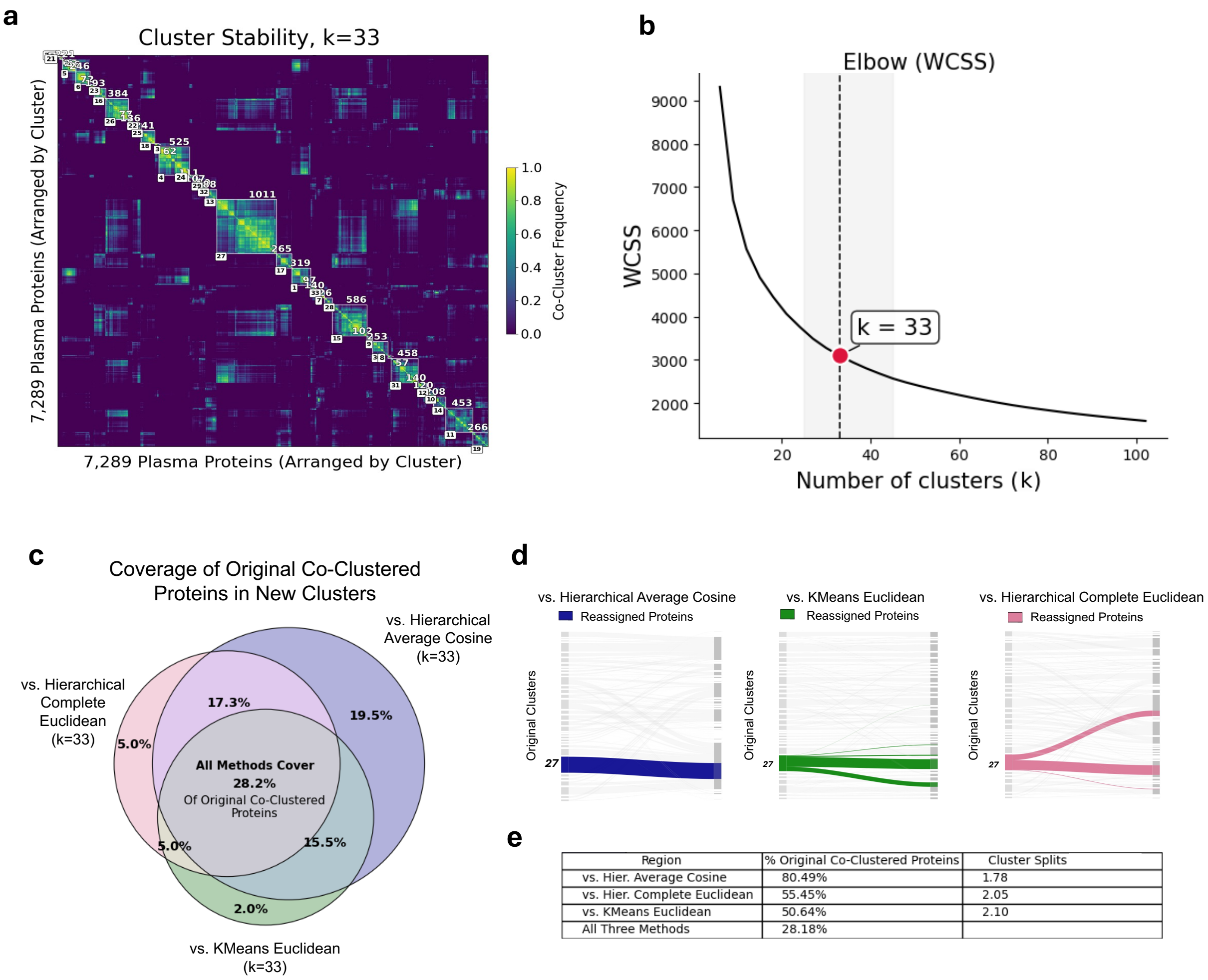

Region	% Original Co-Clustered Proteins	Cluster Splits
vs. Hier. Average Cosine	80.49%	1.78
vs. Hier. Complete Euclidean	55.45%	2.05
vs. KMeans Euclidean	50.64%	2.10
All Three Methods	28.18%	

Supplementary Data Figure 1: *Cluster stability, comparison with other methods, and cluster number rationale.* **a**, Cluster stability analysis (K=33) showing within-cluster consensus across 100 bootstrap resamples. Proteins are ordered by original cluster, outlined in white edged blocks, annotated with cluster number (bottom left label) and size (top right label). Higher values indicate more stable cluster assignments. **b**, Elbow plot of within-cluster sum of squares (WCSS) versus number of clusters. The chosen cluster count k = 33 (red) lies in a plateau indicated with a gray band. **c**, Comparison of clustering methods with k=33 clusters. The percentage overlap of co-occurring proteins within clusters is represented for three alternative methods, with Hierarchical Ward Euclidean as the baseline reference. **d**, Distribution of proteins in original clusters within new clusters for each alternative method, with cluster 27 shown as an example. Hierarchical clustering with average linkage method and cosine distance metric shows the highest retention of original co-clustered protein assignments. The mean size-weighted cluster splits for each original cluster were additionally computed.

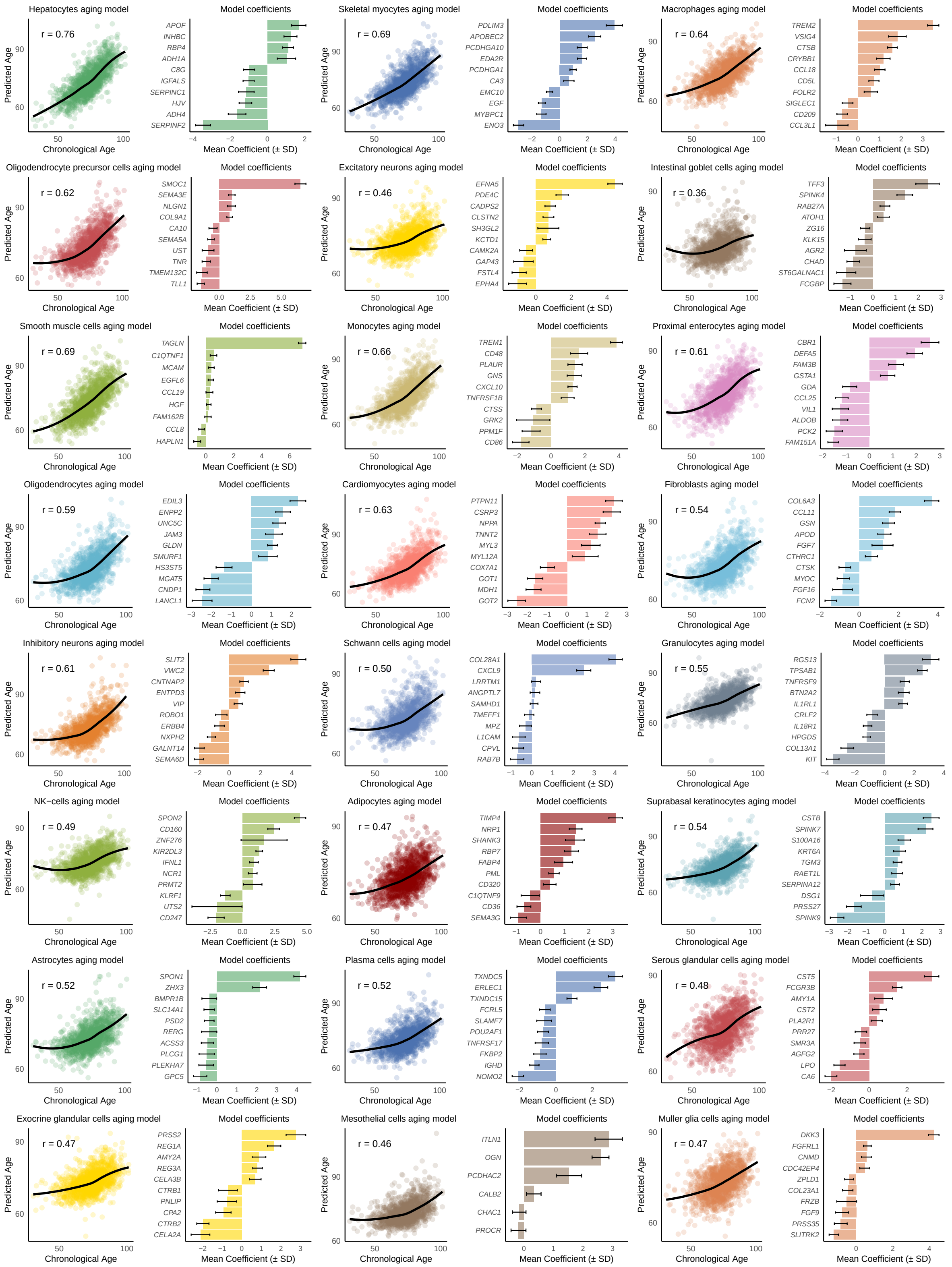

Supplementary Data Figure 2 (Page 1): *Cell type-specific aging model predictions and protein features.* Illustrative cellular aging models trained on healthy individuals in the Knight-ADRC cohort (n=1,398), the largest well-characterized healthy cohort in the GNPC. Scatter plots show estimated biological age versus chronological age with correlation coefficients (r). Bar plots display mean coefficients (± s.d.) of the top proteins by absolute magnitude in each cellular aging model.

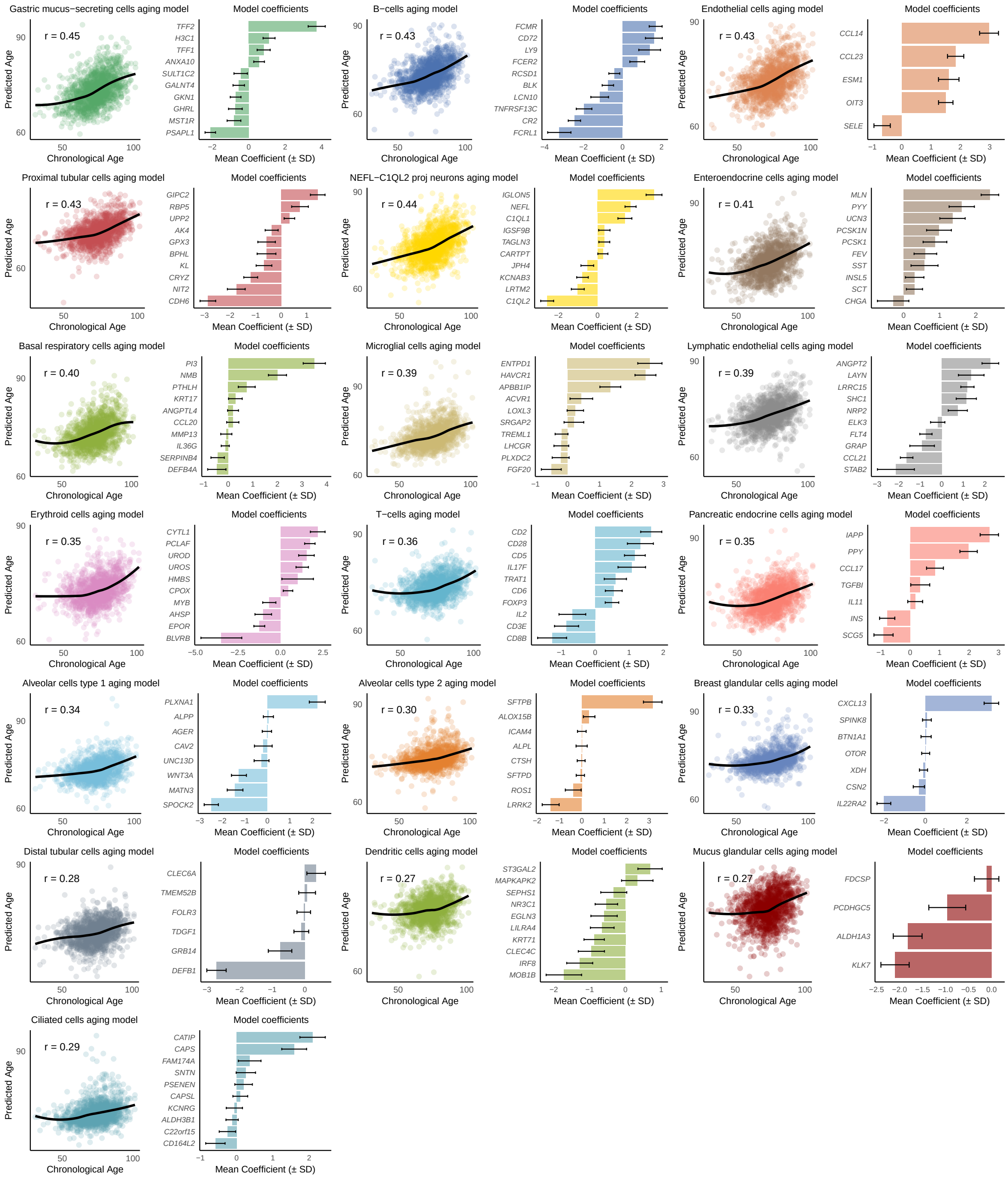

Supplementary Data Figure 2 (Page 2): *Cell type-specific aging model predictions and protein features.* Illustrative cellular aging models trained on healthy individuals in the Knight-ADRC cohort (n=1,398), the largest well-characterized healthy cohort in the GNPC. Scatter plots show estimated biological age versus chronological age with correlation coefficients (r). Bar plots display mean coefficients (± s.d.) of the top proteins by absolute magnitude in each cellular aging model.

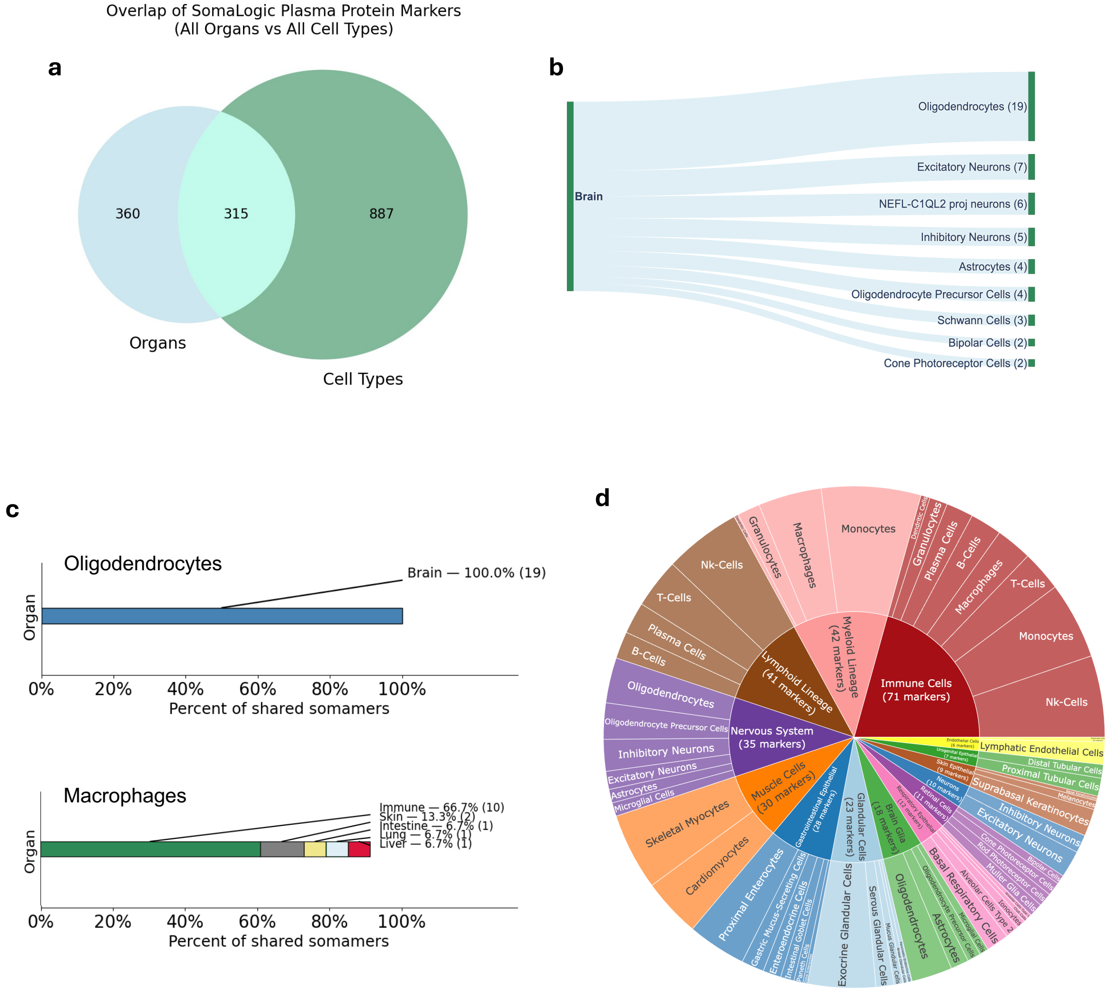

Supplementary Data Figure 3: *Coverage of putative cell-type specific plasma proteins measurable with the SomaScan (7,289 proteins) and Olink (2,923 proteins) proteomics platforms.* **a**, Intersection of putative cellular and organ-enriched plasma proteins measurable with the SomaScan proteomics platform. **b**, Example distribution of organ-enriched plasma proteins (n = 18 organs; brain shown) with respect to individual cell types modeled in the study (n = 60 cell types). Linkages with two or more shared proteins are shown. Organ-enriched proteins were extracted from data provided by Oh et al., 2023[11]. **c**, Intersection of cell-type enriched plasma proteins and organ-enriched plasma proteins measurable with the SomaScan assay. Example distribution of oligodendrocyte and macrophage enriched plasma proteins with respect to organs (percentage Somamers shared). **d**, Intersection of putative lineage and cell-type plasma proteins measurable with the Olink assay. Only proteins measurable by Olink are shown; a complete list of marker genes and corresponding cell-type lineage members is provided in **Supplementary Tables 1, 2**, and **5**

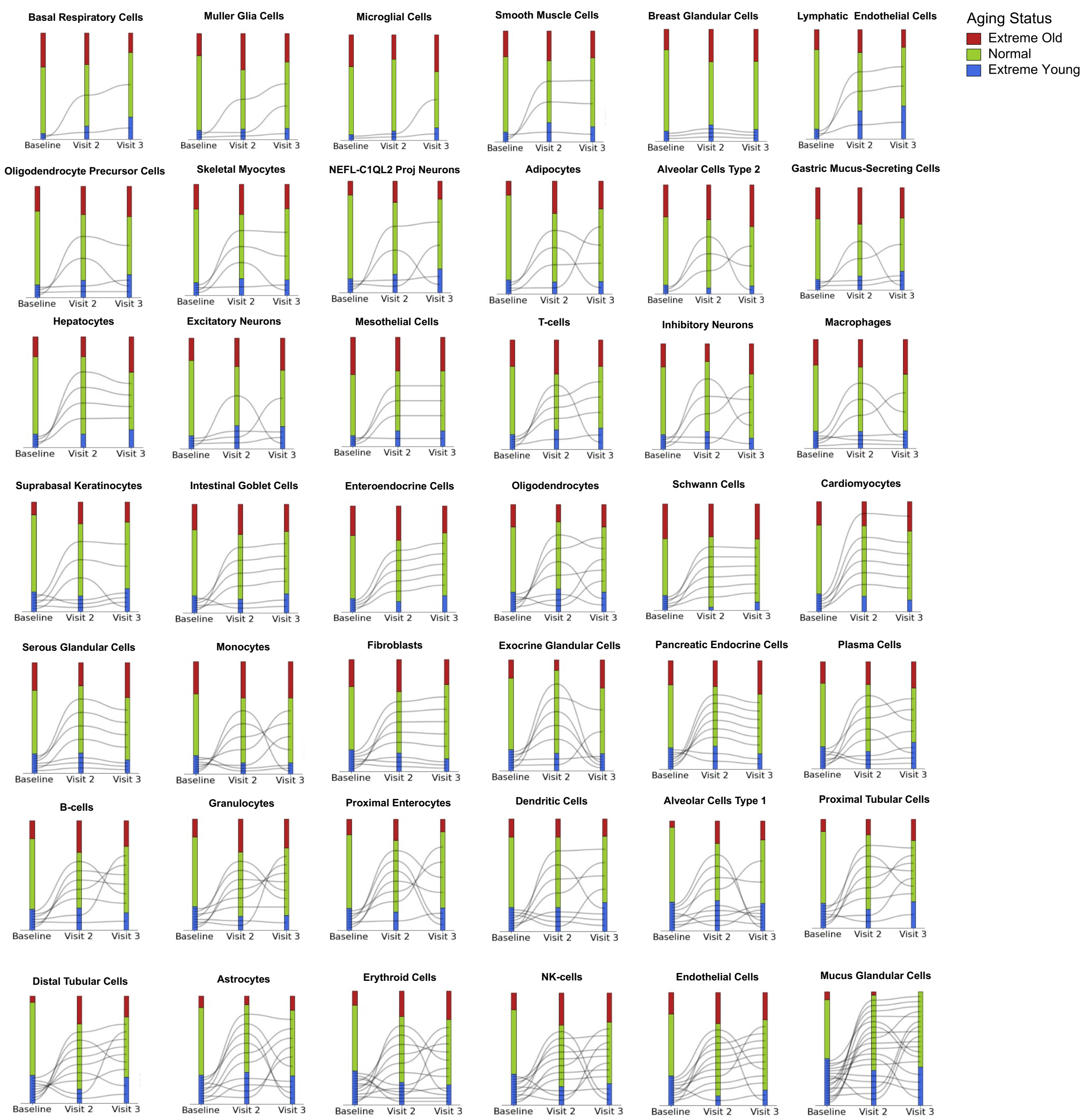

Supplementary Data Figure 4a: *Stability of youthful cell-type aging profiles.* Cell type aging trajectories for 364 individuals in the NSHD 1946 British Birth Cohort across three timepoints for youthful agers at baseline (z-scored age gap <-2). Only cell types with youthful agers present at baseline are shown. Bin heights for extreme agers (youthful and extreme) are scaled relative to the cohort-wide totals at each time point to improve visibility, as these groups represent a small percentage compared to normal agers. Cell-types are plotted in order of increasing number of youthful agers at baseline. Timepoints correspond to longitudinal mean chronological age per blood draw event: baseline: 63.2 $\pm$ 1.1 years; Visit 2 (timepoint 2): 70.7 $\pm$ 0.7 years; Visit 3 (timepoint 3): 72.9 $\pm$ 0.6 years of age. Time points are represented as mean age $\pm$ s.d. (years).

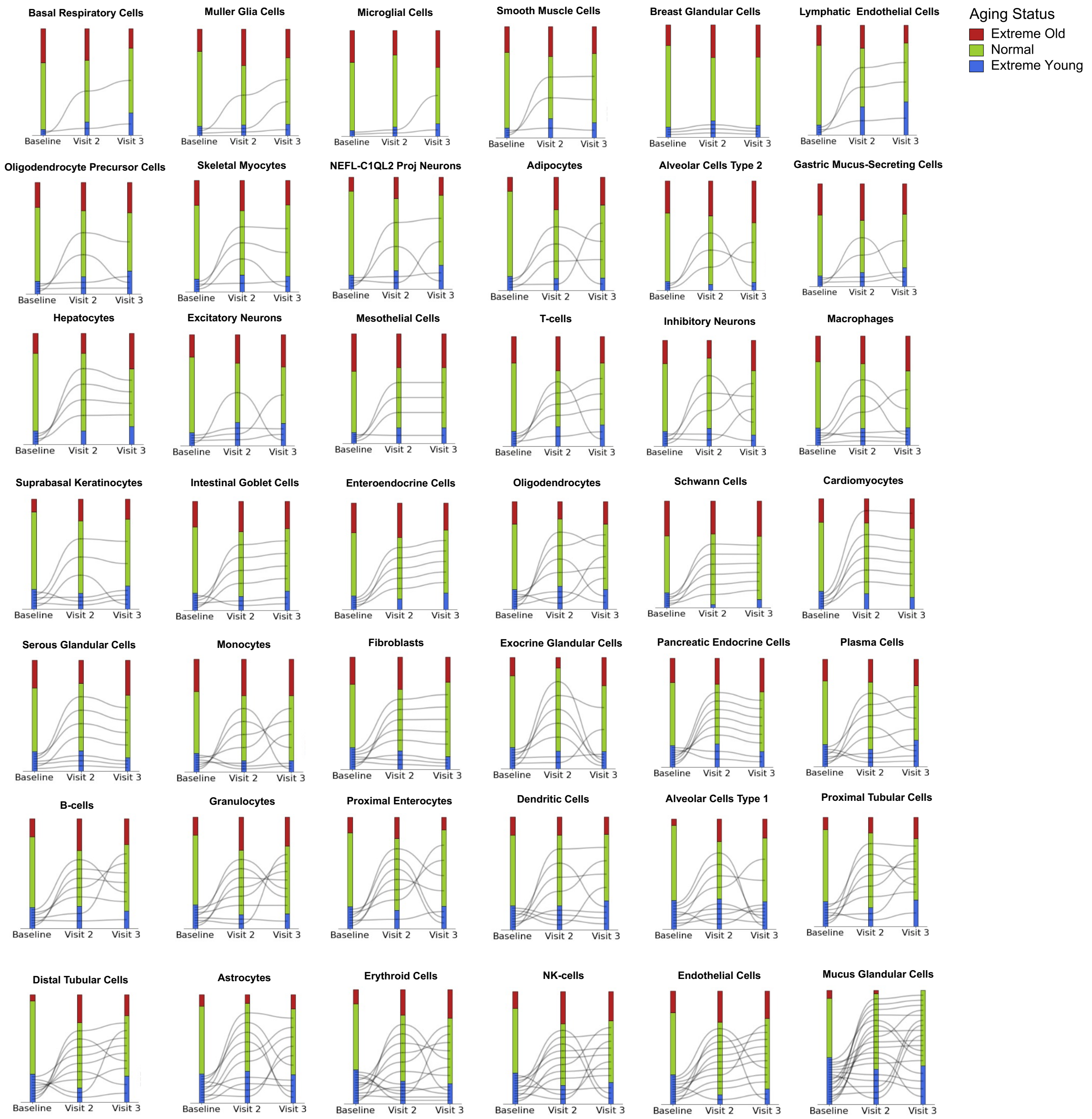

Supplementary Data Figure 4b: *Stability of extreme cell-type aging profiles.* Cell type aging trajectories for 364 individuals in the NSHD 1946 British Birth Cohort across three timepoints for youthful agers at baseline (z-scored age gap <-2). Only cell types with youthful agers present at baseline are shown. Bin heights for extreme agers (youthful and extreme) are scaled relative to the cohort-wide totals at each time point to improve visibility, as these groups represent a small percentage compared to normal agers. Cell-types are plotted in order of increasing number of youthful agers at baseline. Timepoints correspond to longitudinal mean chronological age per blood draw event: baseline: 63.2 $\pm$ 1.1 years; Visit 2 (timepoint 2): 70.7 $\pm$ 0.7 years; Visit 3 (timepoint 3): 72.9 $\pm$ 0.6 years of age. Time points are represented as mean age $\pm$ s.d. (years).

a

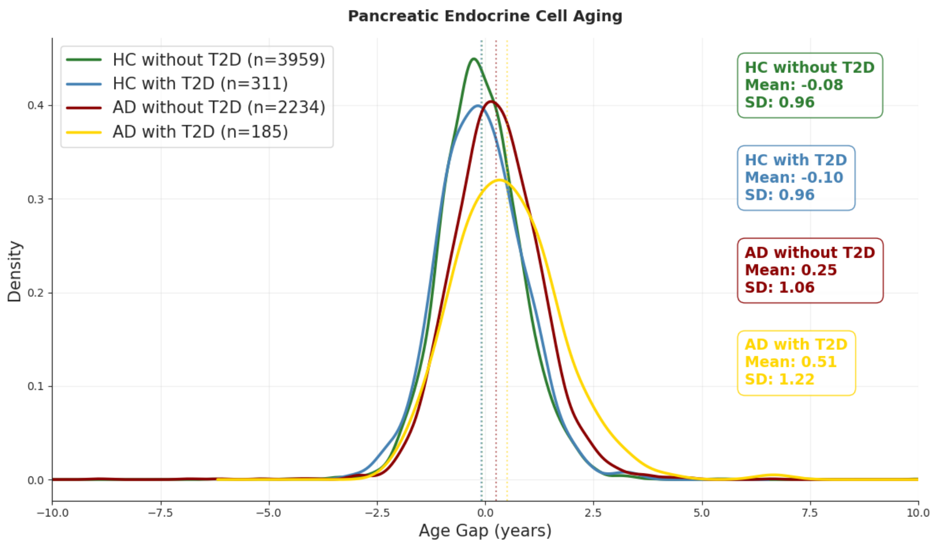

b

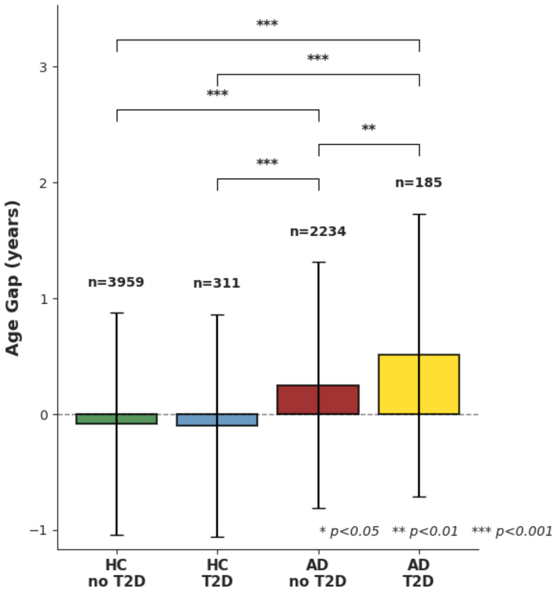

Supplementary Data Figure 5: *Pancreatic endocrine cell aging in Alzheimer's disease (AD) with and without type 2 diabetes comorbidity in the GNPC cohort.* **a**, Density distributions of pancreatic endocrine cell age gaps across four groups: healthy controls (HC) without T2D (n=3959, mean=-0.08, s.d.=0.96), HC with T2D (n=311, mean=-0.10, s.d.=0.96), AD without T2D (n=2234, mean=0.25, s.d.=1.06), and AD with T2D (n=185, mean=0.51, s.d.=1.22). Vertical dashed lines indicate mean values for each group. **b**, Comparison of pancreatic endocrine cell age gaps across groups showing mean (horizontal line) and standard deviation (error bars). Group differences were assessed using two-sided Wilcoxon rank-sum tests. P values are indicated using significance thresholds (*P $<$ 0.05, **P $<$ 0.01, *P $<$ 0.001).

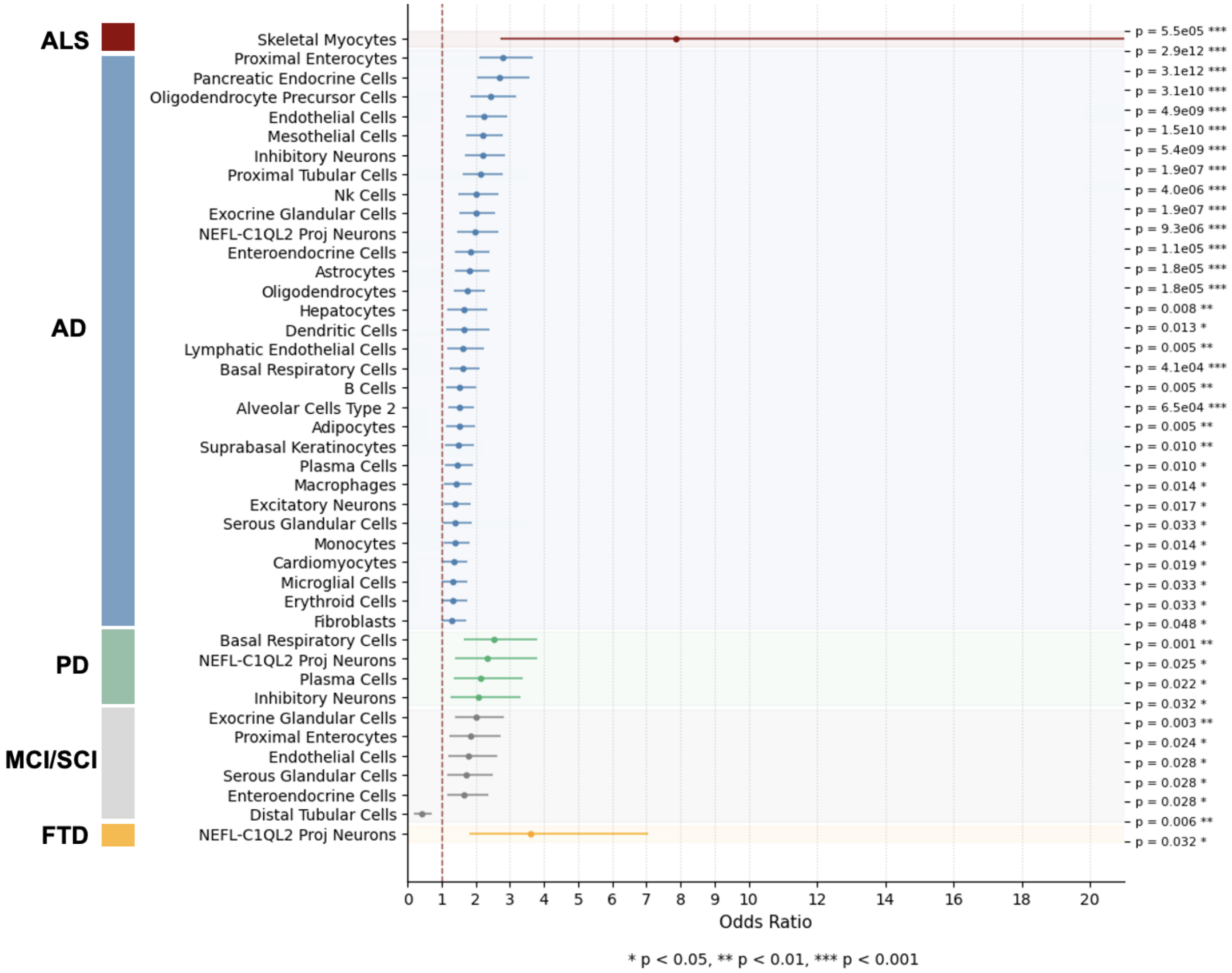

Supplementary Data Figure 6: *Odds ratio analysis of AD, ALS, PD, FTD, and MCI-SCI in the GNPC cohort.* Odds ratios (ORs) and 95% confidence intervals (CIs) for enrichment of extreme-aging outliers across neurodegenerative diseases (AD, ALS, PD, FTD, MCI–SCI) are shown. For each cell type and disease, 2×2 contingency tables were constructed comparing the presence of extreme aging outliers (z-scored age gap > 2) between cases and controls, and two-sided P values were computed using Fisher's exact test. Odds ratios were estimated from these tables, and 95% confidence intervals were calculated using Woolf's method with Haldane-Anscombe correction. P values were adjusted for multiple comparisons using the Benjamini-Hochberg procedure both within each disease and globally across all tests; adjusted P values are reported, and only significant associations are shown.

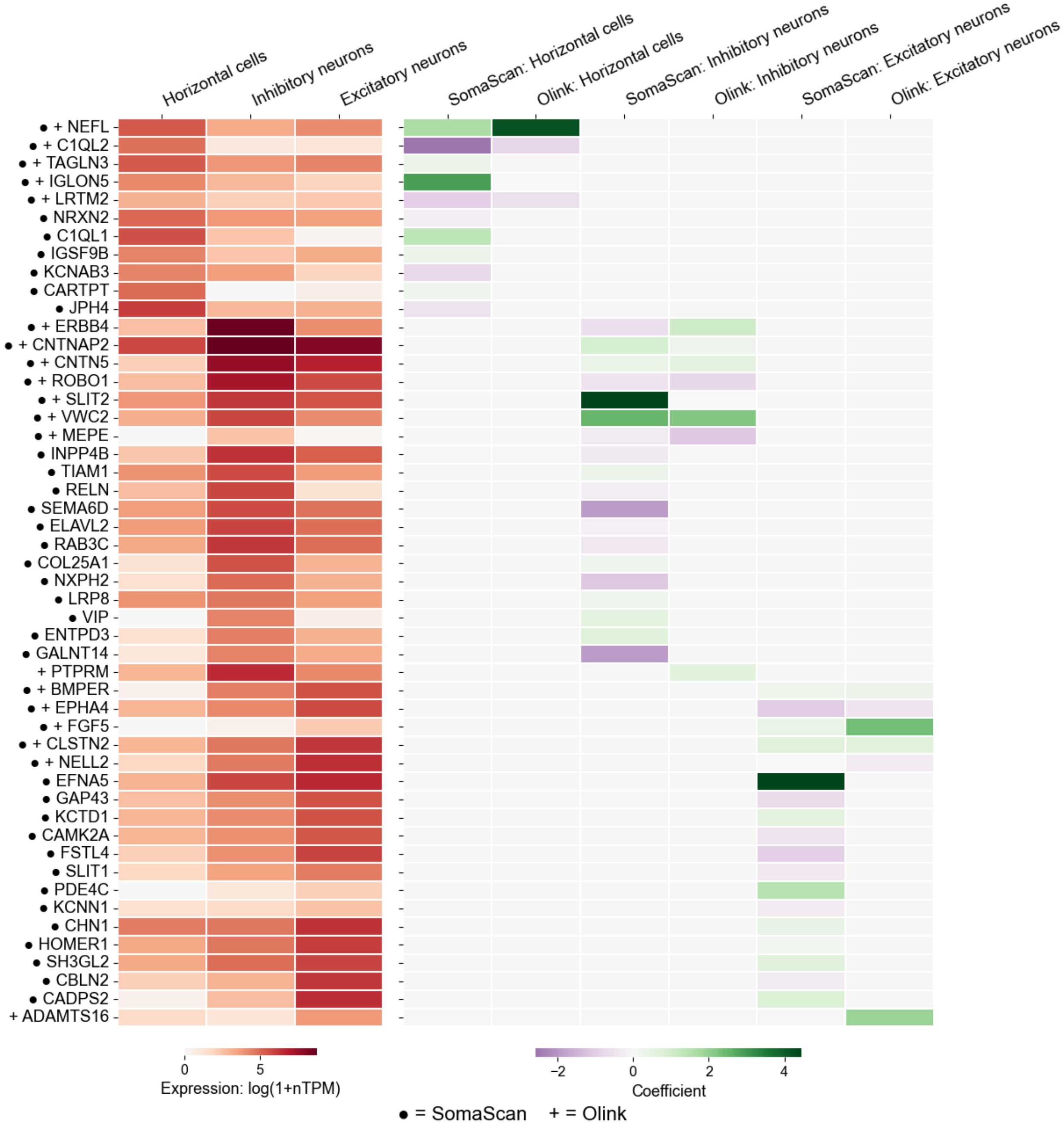

Supplementary Figure 7: *Expression profiles of horizontal cell and neuronal cell signatures and aging model coefficients.* Heatmap displaying gene expression levels from the Human Protein Atlas single-cell transcriptomic dataset (version 24.1) for signatures of horizontal cells, inhibitory neurons, and excitatory neurons. Color intensity represents expression level, with darker red indicating higher expression. Model coefficients from cellular aging clocks for horizontal cells, inhibitory neurons, and excitatory neurons across SomaScan and Olink platforms are shown in adjacent columns. Color hue and intensity reflect coefficient magnitude; only signatures with absolute coefficients > 0.2 are shown. Circles indicate proteins measured with the SomaScan platform, and crosses indicate Olink coverage.

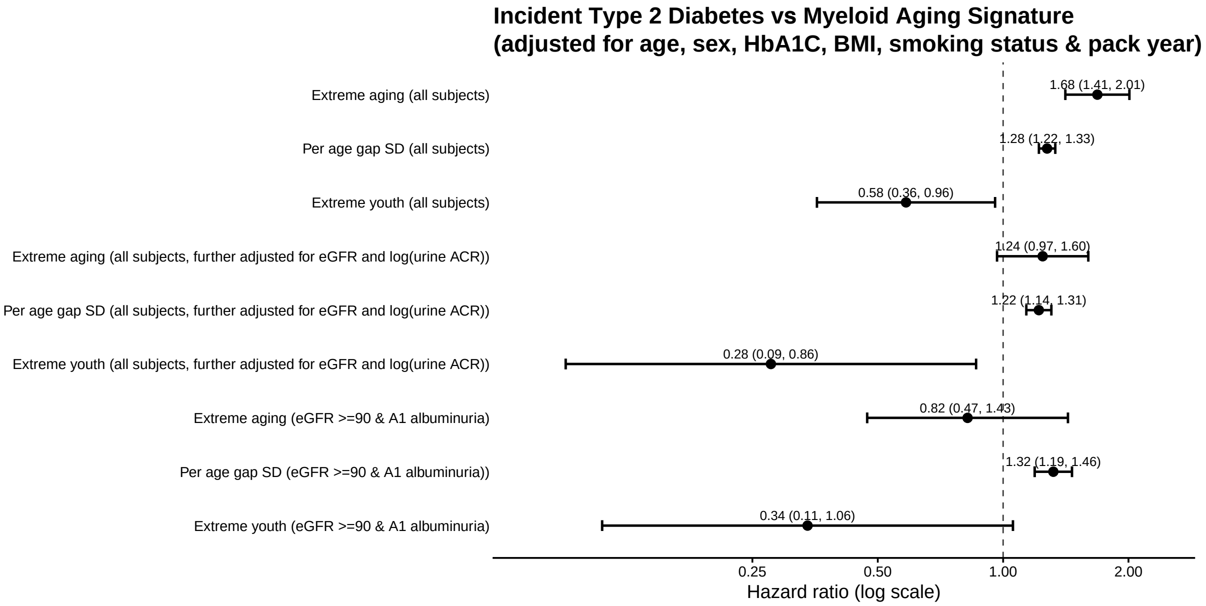

Supplementary Figure 8: *Myeloid cell aging has prognostic power for type 2 diabetes after adjustment for known risk factors (hemoglobin A1c, body mass index, smoking status, pack year history, sex, and age).* Cox proportional hazards models showing hazard ratios (HRs) with 95% confidence intervals (CIs) for z-scored age gap, extreme, and youthful aging. Models were either additionally adjusted for renal function using estimated glomerular filtration rate (eGFR) and $\log(\mathrm{UACR})$, or restricted to individuals without evidence of renal dysfunction, defined as eGFR $\geq 90\,\mathrm{mL\,min^{-1}}\,1.73\,\mathrm{m^{-2}}$ and normal (A1) urine albumin-to-creatinine ratio. Sample sizes were $n = 118$ for individuals with extreme myeloid aging and no renal dysfunction, and $n = 247$ for individuals with extreme myeloid youth and no renal dysfunction.

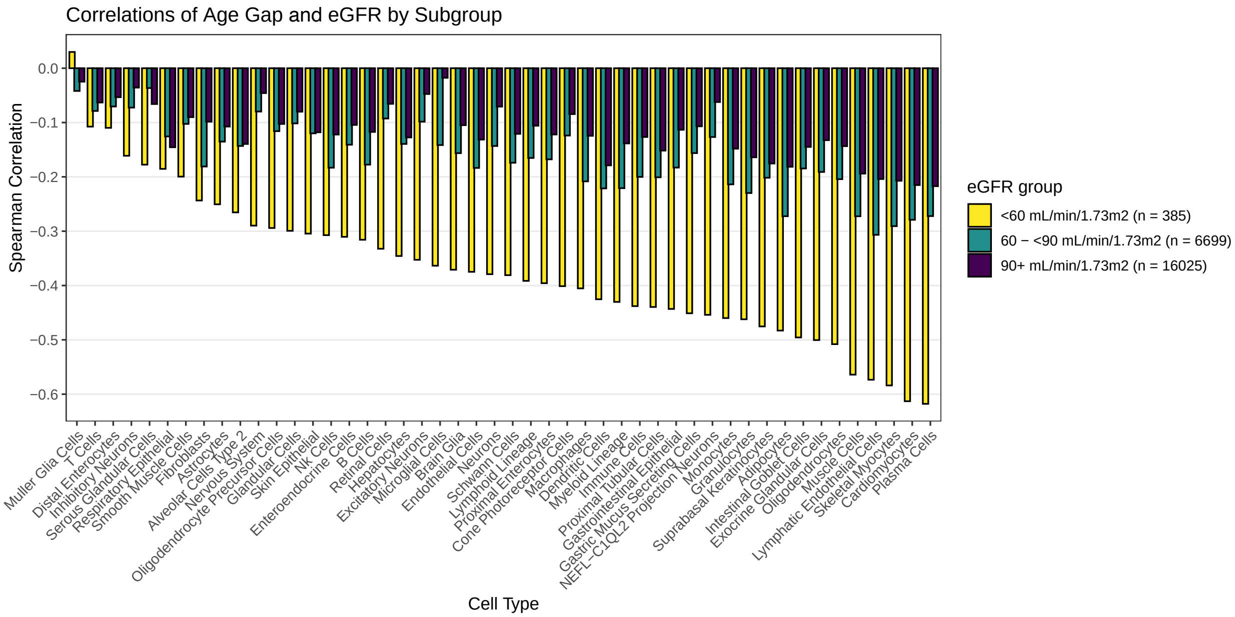

Supplementary Figure 9: *Correlation between age gaps and estimated glomerular filtration rate (eGFR) at normal and reduced eGFR.* Spearman correlation between cellular age gaps and eGFR, stratified by eGFR.

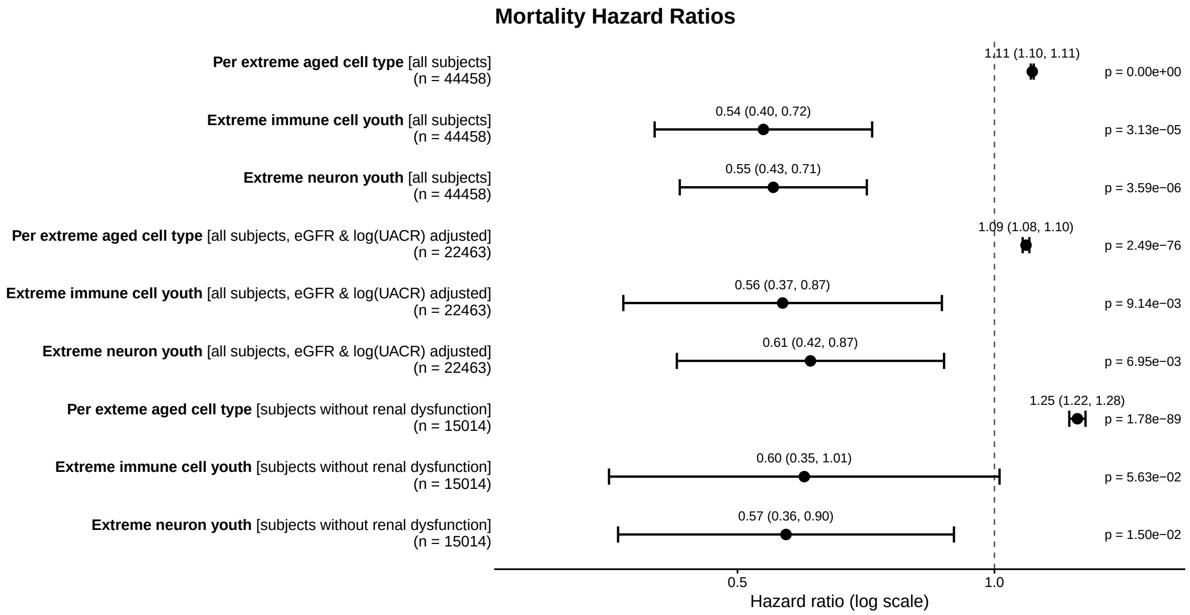

Supplementary Figure 10: *Evaluating the impact of bearing an additional extreme aged cell type on mortality risk and the protective effect associated with youthful aging of immune cells and neurons.* Cox proportional hazards models showing hazard ratios (HRs) with 95% confidence intervals (CIs). All models adjusted for sex and chronological age. Additional adjustment for renal function using estimated glomerular filtration rate (eGFR) and $\log(\mathrm{UACR})$ or restriction to individuals without evidence of renal dysfunction, defined as eGFR $\geq 90\,\mathrm{mL\,min^{-1}}\,1.73\,\mathrm{m^{-2}}$ and normal A1 albuminuria $(\mathrm{UACR} < 30\,\mathrm{mg\,g^{-1}})$ as labeled. Sample sizes are indicated in the plot.

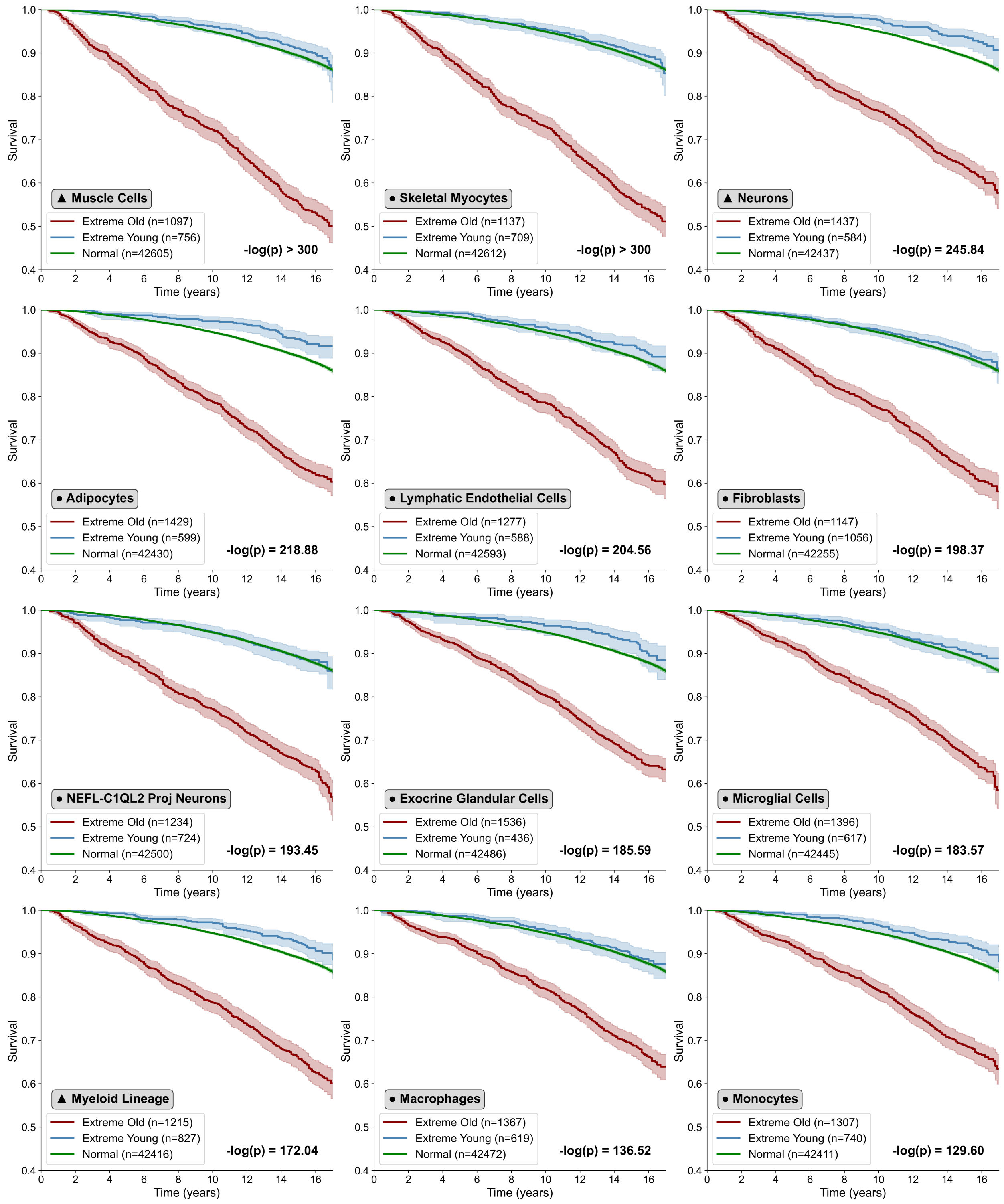

Supplementary Figure 11 (Page 1): *Stratified survival analysis of mortality risk associated with cellular aging.* Kaplan–Meier survival curves showing all-cause mortality stratified by cellular aging status across cell types in the UK Biobank (n = 44,458). Lines represent Kaplan–Meier estimates of survival probability, and shaded bands indicate 95% confidence intervals around the estimated survival function. Participants are categorized into extreme, youthful, and normal aging groups, with sample sizes annotated. Each panel represents a distinct cell type, with circles indicating individual cell types and triangles indicating lineage-level cell types. Group differences in survival were assessed using two-sided log-rank tests. P values were adjusted for multiple comparisons using the Benjamini–Hochberg procedure and are displayed as log10(P).

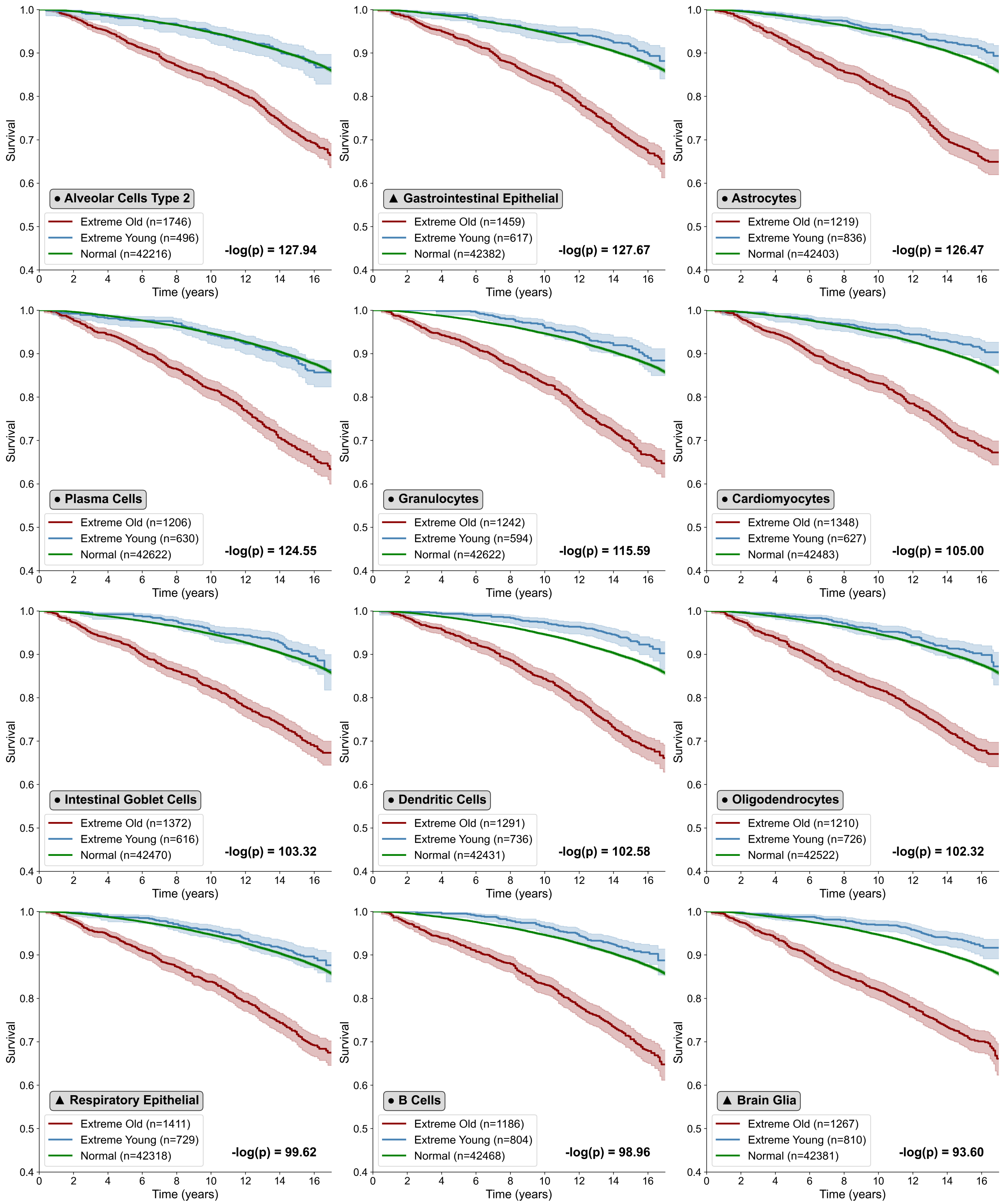

Supplementary Figure 11 (Page 2): *Stratified survival analysis of mortality risk associated with cellular aging.* Kaplan–Meier survival curves showing all-cause mortality stratified by cellular aging status across cell types in the UK Biobank (n = 44,458). Lines represent Kaplan–Meier estimates of survival probability, and shaded bands indicate 95% confidence intervals around the estimated survival function. Participants are categorized into extreme, youthful, and normal aging groups, with sample sizes annotated. Each panel represents a distinct cell type, with circles indicating individual cell types and triangles indicating lineage-level cell types. Group differences in survival were assessed using two-sided log-rank tests. P values were adjusted for multiple comparisons using the Benjamini–Hochberg procedure and are displayed as log10(P).

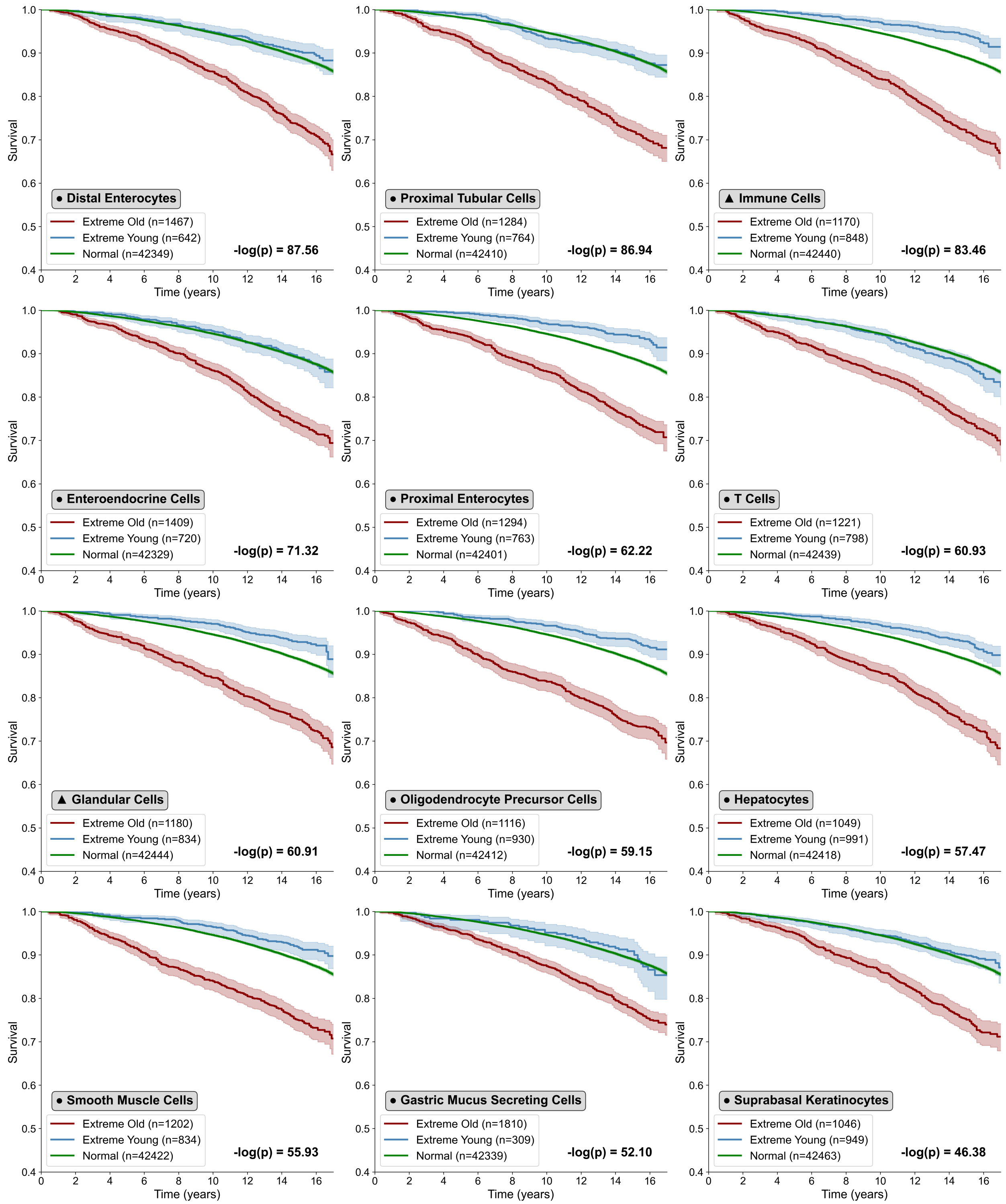

Supplementary Figure 11 (Page 3): *Stratified survival analysis of mortality risk associated with cellular aging.* Kaplan–Meier survival curves showing all-cause mortality stratified by cellular aging status across cell types in the UK Biobank (n = 44,458). Lines represent Kaplan–Meier estimates of survival probability, and shaded bands indicate 95% confidence intervals around the estimated survival function. Participants are categorized into extreme, youthful, and normal aging groups, with sample sizes annotated. Each panel represents a distinct cell type, with circles indicating individual cell types and triangles indicating lineage-level cell types. Group differences in survival were assessed using two-sided log-rank tests. P values were adjusted for multiple comparisons using the Benjamini–Hochberg procedure and are displayed as log10(P).

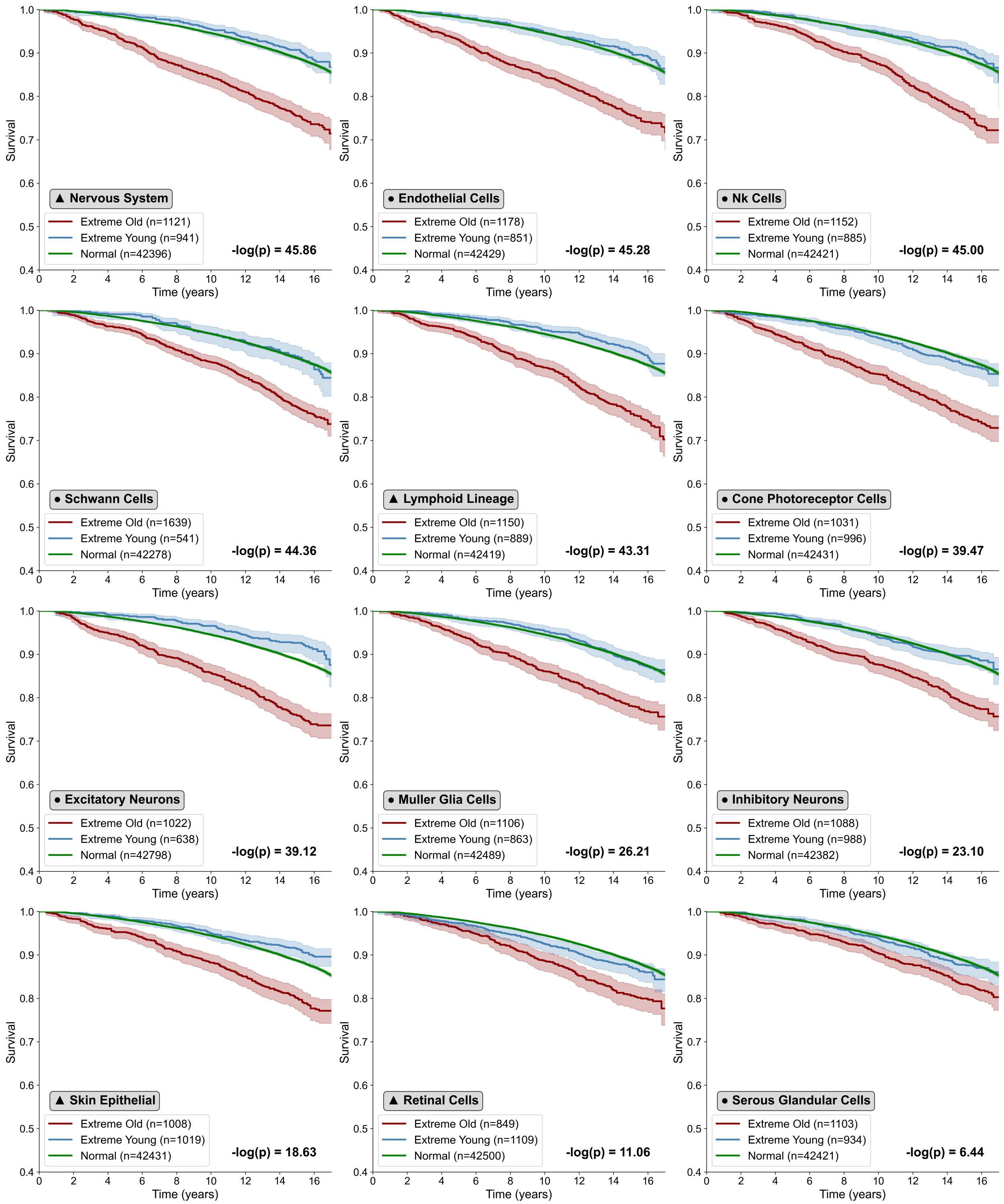

Supplementary Figure 11 (Page 4): *Stratified survival analysis of mortality risk associated with cellular aging.* Kaplan–Meier survival curves showing all-cause mortality stratified by cellular aging status across cell types in the UK Biobank (n = 44,458). Lines represent Kaplan–Meier estimates of survival probability, and shaded bands indicate 95% confidence intervals around the estimated survival function. Participants are categorized into extreme, youthful, and normal aging groups, with sample sizes annotated. Each panel represents a distinct cell type, with circles indicating individual cell types and triangles indicating lineage-level cell types. Group differences in survival were assessed using two-sided log-rank tests. P values were adjusted for multiple comparisons using the Benjamini–Hochberg procedure and are displayed as log10(P).

GNPC V1 Supplemental Member List and Affiliations

- Charles H Adler, Mayo Clinic Arizona, Scottsdale, Arizona, USA
- Alireza Atri, Banner Sun Health Research Institute, Sun City, Arizona, USA
- Thomas G Beach, Banner Sun Health Research Institute, Sun City, Arizona, USA
- Graham Bearden, Alzheimer's Disease Data Initiative, Kirkland, WA
- James D. Berry, Sean M. Healey and AMG Center for ALS, Neurology
- Merce Boada, Ace Alzheimer Center Barcelona, Universitat Internacional de Catalunya, 08029 Barcelona, Spain; Biomedical Research Networking Centre in Neurodegenerative Diseases (CIBERNED), National Institute of Health Carlos III, 28029 Madrid, Spain
- Merle Bode, Hertie Institute for Clinical Brain Research, Neurodegenerative Diseases, Tübingen; German Center of Neurodegenerative Diseases, Department of Neurodegenerative Diseases, Tübingen
- Bradley Boeve, Mayo Clinic, Neurology Department, Rochester, MN
- Hillary Bounds, Gates Ventures, Seattle, WA
- Alfredo Cabrera-Socorro, Johnson & Johnson, NS TA, Beerse, Belgium
- Amanda Fernandez Cano, Ace Alzheimer Center Barcelona, Universitat Internacional de Catalunya, 08029 Barcelona, Spain; Biomedical Research Networking Centre in Neurodegenerative Diseases (CIBERNED), National Institute of Health Carlos III, 28029 Madrid, Spain
- Kaitlin B. Casaletto, University of California, San Francisco, Neurology Department, San Francisco, CA
- Richard J Caselli, Mayo Clinic Arizona, Scottsdale, Arizona, USA
- Yike Chen, Washington University School of Medicine, Department of Psychiatry, St. Louis, 63110, MO, USA.; NeuroGenomics and Informatics Center, Washington University School of Medicine, St. Louis, 63110, MO, USA.
- Matthew H.S. Clement, Alzheimer's Disease Data Initiative, Kirkland, WA
- Eric B. Dammer, Emory University School of Medicine, Atlanta, GA, USA; Emory University School of Medicine, Department of Biochemistry, Atlanta, GA, USA
- Sterre de Boer, Alzheimer Center Amsterdam, Neurology, Amsterdam UMC, Amsterdam, the Netherlands; Amsterdam Neuroscience, Amsterdam, the Netherlands
- Niels De Meirleir, Johnson & Johnson, NS TA, Beerse, Belgium
- Marta del Campo Milan, Barcelonaβeta Brain Research Center (BBRC), Pasqual Maragall Foundation, Barcelona, Spain; Hospital del Mar Research Institute, Barcelona, Spain
- Duc Duong, Emory University School of Medicine, Atlanta, GA, USA; Emory University School of Medicine, Department of Biochemistry, Atlanta, GA, USA
- Maria Victoria Fernandez, Ace Alzheimer Center Barcelona, Universitat Internacional de Catalunya, 08029 Barcelona, Spain
- Lawrence Fourgeaud, Johnson & Johnson, NS TA, La Jolla, USA

- Raquel Puerta Fuentes, Ace Alzheimer Center Barcelona, Universitat Internacional de Catalunya, 08029 Barcelona, Spain; PhD Program in Biotecnology, Faculty of Pharmacy and Food Sciences, University of Barcelona, 08028 Barcelona, Spain
- Jordan Fuller, Gates Ventures, Seattle, WA
- Su Gao, Indiana Alzheimer's Disease Research Center, Indianapolis, IN; Indiana University School of Medicine, Department of Biostatistics & Health Data Science, Indianapolis, IN
- John Gibbons, Rush Alzheimer's Disease Center, Department of Neurological Sciences, Chicago, IL, USA
- Pablo Garcia Gonzalez, Ace Alzheimer Center Barcelona, Universitat Internacional de Catalunya, 08029 Barcelona, Spain; Biomedical Research Networking Centre in Neurodegenerative Diseases (CIBERNED), National Institute of Health Carlos III, 28029 Madrid, Spain
- Gyujin Heo, Washington University School of Medicine, Department of Psychiatry, St. Louis, 63110, MO, USA.; NeuroGenomics and Informatics Center, Washington University School of Medicine, St. Louis, 63110, MO, USA.
- Hilary Heuer, University of California, San Francisco, Neurology Department, San Francisco, CA
- Liping Hou, Johnson & Johnson, Spring House, USA
- Yen-Ning Huang, Indiana Alzheimer's Disease Research Center, Indianapolis, IN; Indiana University School of Medicine, Department of Radiology & Imaging Sciences, Indianapolis, IN
- Clifford R. Jack, Jr, Mayo Clinic, Radiology
- Emily Kogan, Johnson & Johnson, JRD DSDH, Cambridge, USA
- Jessica B Langbaum, Banner Alzheimer's Institute, Phoenix, Arizona, USA
- Argentina Lario-Lago, University of California, San Francisco, Neurology Department, San Francisco, CA
- Shuwei Li, Johnson & Johnson, Spring House, USA
- Shiwei Liu, Indiana Alzheimer's Disease Research Center, Indianapolis, IN; Indiana University School of Medicine, Department of Radiology & Imaging Sciences, Indianapolis, IN
- Menghan Liu, Washington University School of Medicine, Department of Psychiatry, St. Louis, 63110, MO, USA.; NeuroGenomics and Informatics Center, Washington University School of Medicine, St. Louis, 63110, MO, USA.
- Marta Marquie, Ace Alzheimer Center Barcelona, Universitat Internacional de Catalunya, 08029 Barcelona, Spain; Biomedical Research Networking Centre in Neurodegenerative Diseases (CIBERNED), National Institute of Health Carlos III, 28029 Madrid, Spain
- Caitlin P. McHugh, Alzheimer's Disease Data Initiative, Kirkland, WA
- Martine Meyer, Johnson & Johnson, NS TA
- Silke Miller, Johnson & Johnson, NS TA, La Jolla, USA
- Elizabeth Mlynarski, Johnson & Johnson, JRD DSDH, Spring House, USA

- Diederik Moechars, Johnson & Johnson, NS TA, Beerse, Belgium
- Patricia Moran-Losada, Stanford University, The Phil and Penny Knight Initiative for Brain Resilience, Stanford, CA, USA; Stanford University, Wu Tsai Neurosciences Institute, Stanford, CA, USA; Stanford University School of Medicine, Department of Neurology and Neurological Sciences, Stanford, CA, USA
- Paige Opsahl, Gates Ventures, Seattle, WA
- Tamina Park, Indiana Alzheimer's Disease Research Center, Indianapolis, IN; Indiana University School of Medicine, Department of Radiology & Imaging Sciences, Indianapolis, IN
- Mukta Phatak, Alzheimer's Disease Data Initiative, Kirkland, WA
- Joni Lindbohm, MD, PhD, University College London, UCL Brain Sciences, London, UK; University of Helsinki, Clinicum, Helsinki, Finland
- Joseph Pick, Johnson & Johnson, Spring House, USA
- Yolande AL Pijnenburg, Alzheimer Center Amsterdam, Neurology Department, Amsterdam, the Netherlands; Amsterdam Neuroscience, Amsterdam, the Netherlands
- Michael Price, Michael J. Fox Foundation, New York, NY, USA
- Shannon Risacher, Indiana Alzheimer's Disease Research Center, Indianapolis, IN; Indiana University School of Medicine, Department of Radiology & Imaging Sciences, Indianapolis, IN
- Julio C. Rojas, University of California, San Francisco, Neurology Department, San Francisco, CA
- Howard J. Rosen, University of California, San Francisco, Neurology Department, San Francisco, CA
- Tamsin Sargood, Johnson & Johnson, Global Development, UK
- Claudia Schulte, Hertie Institute for Clinical Brain Research, Neurodegenerative Diseases, Tübingen; German Center of Neurodegenerative Diseases , Department of Neurodegenerative Diseases, Tübingen
- Weiwei Schultz, Johnson & Johnson, JRD DSDH, Titusville, USA
- Geidy E Serrano, Banner Sun Health Research Institute, Sun City, Arizona, USA
- Nicholas T. Seyfried, Emory University School of Medicine, Atlanta, GA, USA; Emory University School of Medicine, Department of Neurology, Atlanta, GA, USA; Emory University School of Medicine, Department of Biochemistry, Atlanta, GA, USA
- Todd Sherer, Michael J. Fox Foundation, New York, NY, USA
- Emily Smith, Indiana Alzheimer's Disease Research Center, Indianapolis, IN; Indiana University School of Medicine, Department of Radiology & Imaging Sciences, Indianapolis, IN
- Adam M. Staffaroni, University of California, San Francisco, Neurology Department, San Francisco, CA
- Russell H. Swerdlow, University of Kansas Alzheimer's Disease Research Center, Kansas City, Kansas, USA; University of Kansas, Neurology, Kansas City, Kansas, USA

- Shinya Tasaki, Rush Alzheimer's Disease Center, Department of Neurological Sciences, Chicago, IL, USA
- Charlotte Teunissen, Neurochemistry Laboratory, Neurology Department, Amsterdam, the Netherlands; Amsterdam Neuroscience, Amsterdam, the Netherlands
- Terri G. Thompson, OnPoint Scientific, Inc, San Diego, CA, USA
- Qu Tian, NIH/NIA
- Jigyasha Timsina, Washington University School of Medicine, Department of Psychiatry, St. Louis, 63110, MO, USA.; NeuroGenomics and Informatics Center, Washington University School of Medicine, St. Louis, 63110, MO, USA.
- Abolfazl Doostparast torshizi, Johnson & Johnson, Spring House, USA
- Sergi Valero, Ace Alzheimer Center Barcelona, Universitat Internacional de Catalunya, 08029 Barcelona, Spain; Biomedical Research Networking Centre in Neurodegenerative Diseases (CIBERNED), National Institute of Health Carlos III, 28029 Madrid, Spain
- Wiesje M van der Flier, Alzheimer Center Amsterdam, Neurology Department, Amsterdam, the Netherlands; Amsterdam Neuroscience, Amsterdam, the Netherlands; Epidemiology and Data Science, Amsterdam UMC
- Julia D. Webb, University of California, San Francisco, Neurology Department, San Francisco, CA
- Bryan K Woodruff, Mayo Clinic Arizona, Scottsdale, Arizona, USA
- Ying Xu, Washington University School of Medicine, Department of Psychiatry, St. Louis, 63110, MO, USA.; NeuroGenomics and Informatics Center, Washington University School of Medicine, St. Louis, 63110, MO, USA.
- Mariet A. Younkin, Mayo Clinic, Neurology, Rochester, MN
