# Supplementary material for: Plasma proteomic signatures of cellular aging predict human disease
Source: Nat Med. 2026 Jun 15;32(6):2060–72. doi: 10.1038/s41591-026-04446-y (PMC13279268; doi:10.1038/s41591-026-04446-y)
Supplement: Supplementary file 2 — Reporting Summary [file 41591_2026_4446_MOESM2_ESM.pdf]

nature portfolio

Corresponding author(s): Tony Wyss-Coray

Last updated by author(s): April 14, 2026

Reporting Summary

Nature Portfolio wishes to improve the reproducibility of the work that we publish. This form provides structure for consistency and transparency in reporting. For further information on Nature Portfolio policies, see our Editorial Policies and the Editorial Policy Checklist.

Statistics

For all statistical analyses, confirm that the following items are present in the figure legend, table legend, main text, or Methods section.

n/a	Confirmed	
☐	☒	The exact sample size (n) for each experimental group/condition, given as a discrete number and unit of measurement
☐	☒	A statement on whether measurements were taken from distinct samples or whether the same sample was measured repeatedly
☐	☒	The statistical test(s) used AND whether they are one- or two-sided<br>*Only common tests should be described solely by name; describe more complex techniques in the Methods section.*
☐	☒	A description of all covariates tested
☐	☒	A description of any assumptions or corrections, such as tests of normality and adjustment for multiple comparisons
☐	☒	A full description of the statistical parameters including central tendency (e.g. means) or other basic estimates (e.g. regression coefficient) AND variation (e.g. standard deviation) or associated estimates of uncertainty (e.g. confidence intervals)
☐	☒	For null hypothesis testing, the test statistic (e.g. F, t, r) with confidence intervals, effect sizes, degrees of freedom and P value noted<br>*Give P values as exact values whenever suitable.*
☒	☐	For Bayesian analysis, information on the choice of priors and Markov chain Monte Carlo settings
☒	☐	For hierarchical and complex designs, identification of the appropriate level for tests and full reporting of outcomes
☐	☒	Estimates of effect sizes (e.g. Cohen's d, Pearson's r), indicating how they were calculated

Our web collection on statistics for biologists contains articles on many of the points above.

Software and code

Policy information about availability of computer code

Data collection: All data utilized in this study are obtained from previously published studies and have been processed in their data collection process, so no software tool was used for this purpose in our work.

Data analysis: All analyses were performed using freely available packages in Python (v3.10.16 or v3.11.6) and R v4.1.2

Python v3.10.16 (analyses in the UK Biobank and NSHD datasets):
pandas==2.2.3
numpy==1.26.4
seaborn==0.13.2
matplotlib==3.10.0
sklearn==1.5.2
statsmodels==0.14.4
scipy==1.15.1
gprofiler-official==1.0.0

Python v3.11.6 (all analyses in the GNPC dataset):
pandas==2.1.1
numpy==1.24.4
seaborn==0.13.0
matplotlib==3.8.0
sklearn==1.3.1

statsmodels==0.14.0, scipy==1.11.3 gprofiler-official==1.0.0

R v4.I.2 (analyses in NSHD, and clock development in the UK Biobank and GNPC)
glmnet==4.1.7
ggplot2==3.4.2 , survival==3.4.2

For manuscripts utilizing custom algorithms or software that are central to the research but not yet described in published literature, software must be made available to editors and reviewers. We strongly encourage code deposition in a community repository (e.g. GitHub). See the Nature Portfolio guidelines for submitting code & software for further information.

Data

Policy information about availability of data

All manuscripts must include a data availability statement. This statement should provide the following information, where applicable:

- Accession codes, unique identifiers, or web links for publicly available datasets
- A description of any restrictions on data availability
- For clinical datasets or third party data, please ensure that the statement adheres to our policy

GNPC data is available upon request to qualified researchers through a standard protocol (https://www.neuroproteome.org/harmonized-data-set-hds). Access is contingent on adherence to the GNPC Data Use Agreement and the Publication Policies. The Knight-ADRC proteomics data were generated by the laboratory of C.C. (cruchagac@wustl.edu) and can be requested at https://knightadrc.wustl.edu/professionals-clinicians/request-center-resources/submit-a-request. UK Biobank data are available upon request through a standard protocol (https://www.ukbiobank.ac.uk/register-apply). Bona fide researchers can apply to access the NSHD data via a standard application procedure (further details available at https://skylark.ucl.ac.uk/NSHD/access/). Mortality data can be requested from the UK Longitudinal Linkage Collaboration (https://ukllc.ac.uk/). Access to controlled datasets requires submission of a formal application and data use agreement; requests are reviewed by the relevant data access committees and typically receive a response within approximately 4–12 weeks.

Research involving human participants, their data, or biological material

Policy information about studies with human participants or human data. See also policy information about sex, gender (identity/presentation), and sexual orientation and race, ethnicity and racism.

Reporting on sex and gender	Self-reported sex labels were used as a covariate in cellular aging model training, Cox proportional hazards models, and linear regression analyses. Sex-stratified analyses were performed for disease risk and all-cause mortality associations.
Reporting on race, ethnicity, or other socially relevant groupings	We did not utilize race or ethnicity in any analyses in this study.
Population characteristics	The study included three cohorts: (1) GNPC (n=14,281 from 14 independent cohorts; 7,074 healthy controls and subpopulations with AD [n=2,761], ALS [n=245], PD [n=476], FTD [n=199], and MCI-SCI [n=1,992]); (2) NSHD 1946 British Birth Cohort (n=1,803 at baseline, with 364 individuals followed longitudinally across three timepoints and 483 in the Insight-46 neuroimaging substudy); and (3) UK Biobank (n=44,458 after quality control, aged 40-69 at recruitment).
Recruitment	Participant recruitment for each cohort is detailed in the Methods section and corresponding primary publications. All cohorts represent existing resources with data available upon request through their respective data access procedures.
Ethics oversight	All cohorts and studies were approved by their respective ethics boards.

Note that full information on the approval of the study protocol must also be provided in the manuscript.

Field-specific reporting

Please select the one below that is the best fit for your research. If you are not sure, read the appropriate sections before making your selection.

☒ Life sciences ☐ Behavioural & social sciences ☐ Ecological, evolutionary & environmental sciences

For a reference copy of the document with all sections, see nature.com/documents/nr-reporting-summary-flat.pdf

Life sciences study design

All studies must disclose on these points even when the disclosure is negative.

Sample size	Sample size was determined by the number of participants with completely reported plasma proteomics data. No other sample size calculations were performed, but sample sizes are comparable to prior proteomics studies involving human cohorts.
Data exclusions	Individuals with incomplete reporting of age and or sex were excluded from the study.
Replication	No replication was performed in the context of life science study design, as no experimental data was produced in our study. The computational analyses and their results are fully reproducible given the same input data and specified random seed.

Randomization	All data utilized in this manuscript are obtained from previously published studies. Randomization was not applicable as our study focused on computational analysis of these existing datasets.
Blinding	All data utilized in this manuscript are obtained from previously published studies. All the analyses were performed systematically regardless of sample identity, so the process does not involve potential experimental bias.

Reporting for specific materials, systems and methods

We require information from authors about some types of materials, experimental systems and methods used in many studies. Here, indicate whether each material, system or method listed is relevant to your study. If you are not sure if a list item applies to your research, read the appropriate section before selecting a response.

Materials & experimental systems

n/a	Involved in the study
☒	☐ Antibodies
☒	☐ Eukaryotic cell lines
☒	☐ Palaeontology and archaeology
☒	☐ Animals and other organisms
☒	☐ Clinical data
☒	☐ Dual use research of concern
☒	☐ Plants

Methods

n/a	Involved in the study
☒	☐ ChIP-seq
☒	☐ Flow cytometry
☒	☐ MRI-based neuroimaging

Plants

Seed stocks	*Report on the source of all seed stocks or other plant material used. If applicable, state the seed stock centre and catalogue number. If plant specimens were collected from the field, describe the collection location, date and sampling procedures.*
Novel plant genotypes	*Describe the methods by which all novel plant genotypes were produced. This includes those generated by transgenic approaches, gene editing, chemical/radiation-based mutagenesis and hybridization. For transgenic lines, describe the transformation method, the number of independent lines analyzed and the generation upon which experiments were performed. For gene-edited lines, describe the editor used, the endogenous sequence targeted for editing, the targeting guide RNA sequence (if applicable) and how the editor was applied.*
Authentication	*Describe any authentication procedures for each seed stock used or novel genotype generated. Describe any experiments used to assess the effect of a mutation and, where applicable, how potential secondary effects (e.g. second site T-DNA insertions, mosiacism, off-target gene editing) were examined.*
